# π covalency in the halogen bond

**DOI:** 10.1038/s41467-020-17122-7

**Published:** 2020-07-03

**Authors:** Cameron W. Kellett, Pierre Kennepohl, Curtis P. Berlinguette

**Affiliations:** 1 Department of Chemistry, The University of British Columbia, 2036 Main Mall, Vancouver, BC V6T 1Z1 Canada; 2 Department of Chemical and Biological Engineering, The University of British Columbia, 2360 East Mall, Vancouver, BC V6T 1Z3 Canada; 3 Stewart Blusson Quantum Matter Institute, The University of British Columbia, 2355 East Mall, Vancouver, BC V6T 1Z4 Canada; 4 Canadian Institute for Advanced Research (CIFAR), 661 University Avenue, Toronto, ON M5G 1M1 Canada

**Keywords:** Physical chemistry, Halogen bonding, Density functional theory

## Abstract

Halogen bonds are a highly directional class of intermolecular interactions widely employed in chemistry and chemical biology. This linear interaction is commonly viewed to be analogous to the hydrogen bond because hydrogen bonding models also intuitively describe the σ-symmetric component of halogen bonding. The possibility of π-covalency in a halogen bond is not contemplated in any known models. Here we present evidence of π-covalency being operative in halogen bonds formed between chloride and halogenated triphenylamine-based radical cations. We reach this conclusion through computational analysis of chlorine K-edge X-ray absorption spectra recorded on these halogen bonded pairs. In light of this result, we contend that halogen bonding is better described by analogy to metal coordination bonds rather than hydrogen bonds. Our revised description of the halogen bond suggests that these interactions could be employed to influence the electronic properties of conjugated molecules in unique ways.

## Abstract (web summary)

Current models of halogen bonding describe the σ-symmetric component of this interaction but do not contemplate the possibility of π-covalency. Here the authors provide experimental and computational evidence of π-covalency in halogen bonds involving radical cation halogen bond donors.

## Introduction

Halogen bonding (XB) is a strong intermolecular interaction that is commonly compared to hydrogen bonding (HB)^1–7^. Both interactions are nearly linear attractions between a nucleophile (an HB or XB-acceptor) and an electrophilic site on the terminus of either a hydrogen or halogen atom bonded to an electron-withdrawing group (an HB or XB-donor, respectively)^5–8^. Together, they comprise some of the strongest known intermolecular interactions^6,9,10^. Halogen bonding is widely used in many of the same applications as hydrogen bonding, including organic synthesis^11^, crystal engineering^7,12^, polymer engineering^13^, and medicinal chemistry^14,15^.

**Fig. 1:**
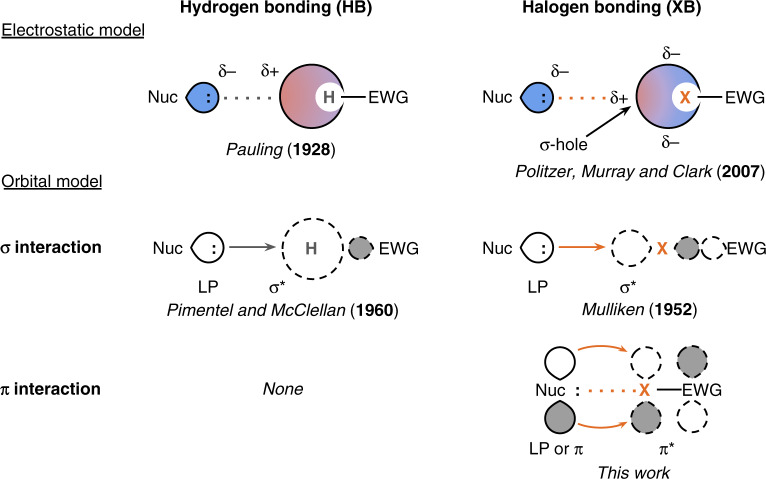
Hydrogen bonding and halogen bonding models. Schematic representations of the electrostatic and covalent charge-transfer models of hydrogen bonding and halogen bonding^8,16,17,21,22,28,29^. Our study shows that covalent *π* bonding can also be operative in halogen bonds.

Halogen bonding is often described in the same terms as hydrogen bonding. The most commonly cited models of both interactions focus on the electrostatic attraction between the partial negative charge of a nucleophile with either the partial positive charge of a hydrogen atom or a small region of partial positive charge on the terminus of a halogen atom known as a σ-hole (Fig. 1)^16–20^. Other descriptions focus on the covalent charge-transfer component, where the lone pair of the nucleophile donates electron density into the hydrogen or halogen σ* antibonding orbital of the HB or XB-donor^4,8,21–29^. While both of these models have advantages and disadvantages, and an expansive view of these interactions will consider both as contributing factors^7,21,24,30–33^, they both describe hydrogen bonding and halogen bonding as the same type of interaction.

Despite these strong parallels between hydrogen bonding and halogen bonding, this analogy overlooks the possibility that halogen bonds can mediate not just σ-symmetric charge transfer, but also π-symmetric charge transfer (Fig. 1). While an HB-donor only has σ-symmetric orbitals on hydrogen, an XB-donor also has orbitals perpendicular to the σ-bond axis that are ideally oriented for π-symmetric orbital overlap. An intermolecular π-bond is therefore conceivable in cases where complementary low-energy empty π* orbitals and high-energy filled π orbitals are present on the XB-donor and acceptor. As an example, consider a halogen bond formed with a π-donating nucleophile. In many if not most cases, a π-covalent interaction will not occur because all energetically accessible π orbitals are filled (Supplementary Fig. 1a). However, π-covalency is possible when low-energy empty π orbitals on the XB-donor are made available, either by removing an electron from the filled π orbital (Supplementary Fig. 1b) or by lowering the energy of the π* orbital (Supplementary Fig. 1c). The opposite situation, where π backdonation occurs from the XB-donor to the XB-acceptor, is also plausible with appropriately positioned π and π* orbitals.

We therefore propose that metal–ligand coordination bonds, where π-symmetric dative interactions are well known, make a better analogy for the nature of halogen bonding than hydrogen bonds. This shift in thinking has practical implications in addition to just rhetorical ones. It is well known that π-interactions in metal–ligand bonds have dramatic effects on the electronic properties of π-conjugated ligands. By extension, we believe that targeted design of halogen bonding pairs could leverage π-interactions to similarly impact the electronic properties of π-conjugated XB-donors and acceptors. Theoretical work by the Wong group has suggested that π backdonation contributes to the strength of halogen bonds formed with nitroxide radical (R_2_NO^•^) nucleophiles^34^, but experimental validation and widespread discussion of the possibility of π-covalency in halogen bonds are completely absent in the literature.

**Fig. 2:**
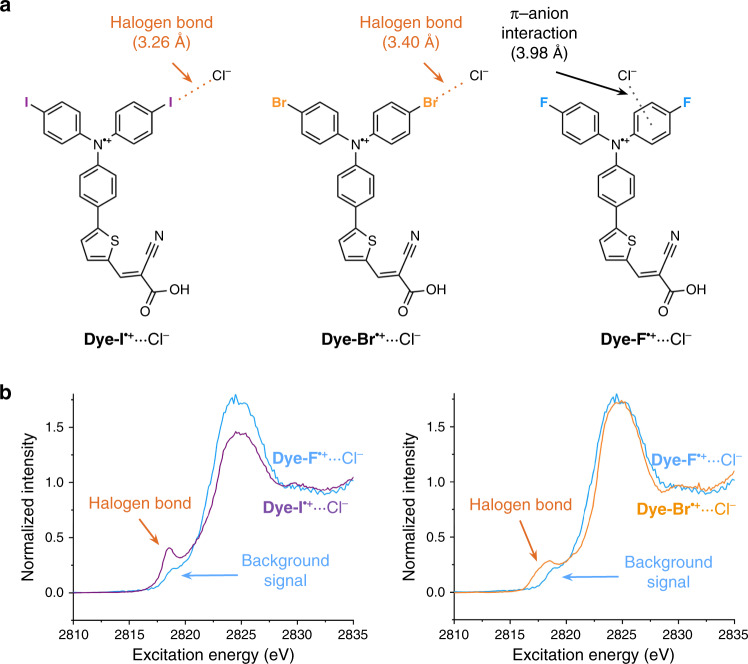
Predicted adduct structures and experimental XAS spectra. **a** The **Dye-X**^**•+**^···Cl^−^ adduct structures predicted by DFT methods. **b** The experimental XAS spectra of the **Dye-I**^**•+**^···Cl^−^ halogen bonded dimer (purple line) and the **Dye-Br**^**•+**^···Cl^−^ halogen bonded dimer (orange line) overlaid with the non-halogen bonding **Dye-F**^**•+**^···Cl^−^ control (blue line)^37^.

We provide herein an analysis of experimental data that points to the clear existence of π-covalency in a halogen bond. We experimentally resolve this π-covalent component by studying a homologous pair of triphenylamine-based XB-donors denoted as **Dye-X** (where X = Br or I), along with the non-halogen bonding analog, **Dye-F** (Fig. 2a)^35–37^. When oxidized by one electron to form the corresponding radical cation, the singly occupied molecular orbital (SOMO) of the open-shell **Dye-X**^**•+**^ is a π-symmetric MO with lobes oriented orthogonally to the halogen bond axis^36^. This SOMO is ideally positioned both spatially and energetically to accept electron density from a π-donating nucleophile as illustrated in Supplementary Fig. 1b. K-edge X-ray absorption spectroscopy (XAS) is a powerful tool to investigate the electronic structure of metal–ligand bonds^38–40^. For halogen bonded dimers featuring a chloride nucleophile, it is possible to use chlorine XAS to probe the electronic structure of halogen bonds^41,42^. For the halogen bonded chloride adducts of **Dye-I**^**•+**^ and **Dye-Br**^**•+**^, we find that the well-resolved low-energy “pre-edge” spectral signature arises from π-orbital covalency.

## Results

### Experimental design

The **Dye-X** compounds consist of a redox-active triphenylamine core decorated with halogen substituents and an anchoring group to adhere the compounds to a metal oxide surface (Fig. 2a). The triphenylamine moiety is not an electron-withdrawing group in the neutral form and consequently does not facilitate significant halogen bonding interactions. One-electron oxidation of the molecules to the corresponding radical cation states **Dye-I**^**•+**^ and **Dye-Br**^**•+**^ greatly increases the electronegativity of the triphenylamine core and thereby promotes halogen bonding^37^. The triphenylamine core stabilizes the radical cation sufficiently to allow for spectroscopic studies^43,44^. The **Dye-X** compounds were adsorbed to a solid mesoporous TiO_2_ thin film with a common molecular orientation (Supplementary Fig. 2), thereby facilitating the study of halogen bonding interactions with a solution-phase species while suppressing self-interaction between **Dye-X** molecules^45^. For our XAS experiments, the surface-anchored **Dye-X** compounds were oxidized with an acetonitrile solution of nitrosonium tetrafluoroborate (NOBF_4_) then immersed in an acetonitrile solution of tetrabutylammonium chloride (NBu_4_Cl) and frozen at 77 K^37^.

### Experimental chlorine K-edge X-ray absorption spectra

K-edge XAS directly probes the covalent interactions of individual elements in a molecular system by selectively exciting the core 1s electrons with high-energy X-rays. This technique made it possible for us to isolate and selectively excite transitions in the **Dye-X**^**•+**^···Cl^–^ dimers that involve only the nucleophilic chloride ions. A schematic describing the origins of the major XAS spectral features is presented in Supplementary Fig. 3. In the absence of this covalent charge-transfer interaction, the first available Cl K-edge XAS transition is the excitation from the 1s shell into the empty Cl 4p orbitals of all chloride atoms present in the sample. This is observed as a very intense edge peak. Crucial to this study is the fact that a well-resolved pre-edge peak is observed if the valence 3p electrons of a Cl^–^ ion form a covalent interaction with the empty orbitals on the halogen of a **Dye-X**^**•+**^ molecule. The integrated intensity of the pre-edge peak is directly proportional to the degree of orbital mixing in this covalent interaction^39,46^.

The experimental Cl K-edge XAS spectra of the halogen bonded **Dye-I**^**•+**^···Cl^–^ and **Dye-Br**^**•+**^···Cl^–^ dimers are shown in Fig. 2b^37^. Three main spectroscopic features are observed prior to the edge jump at >2828 eV: A well-resolved pre-edge peak near 2818 eV; a pre-edge shoulder in the 2820–2 eV range; and an intense edge peak between 2822 and 2828 eV. These features are highlighted using first and second derivative analyses in Supplementary Fig. 4. The pre-edge peak (~2818 eV) has a greater integrated area for **Dye-Br**^**•+**^···Cl^–^ than for **Dye-I**^**•+**^···Cl^–^, whilst the inverse is true of the pre-edge shoulder (2820–2 eV). In our previous study, we assigned the well-resolved pre-edge peak as an excitation into the σ* orbital of the C–X bond in accordance with the previously reported literature^37^. In this present study we will demonstrate that our preliminary assignment was indeed incorrect, and that the pre-edge transition is better described as an excitation into the π* orbitals of **Dye-X**^**•+**^.

The Cl K-edge XAS experiment does not discriminate between Cl atoms in different chemical environments, and therefore the observed spectra are a superposition of signals from all Cl atoms present in the sample. Moreover, the experimental XAS spectra in our previous study were collected in the presence of an excess of Cl^–^, and therefore the XAS signals arising from XB interactions will necessarily be convoluted by signals arising from other intermolecular interactions—most notably π-anion interactions between Cl^–^ and the triphenylamine group of **Dye-X**^**•+**^—as well as interactions between the Cl^–^ ions and the TiO_2_ substrate. The fluorinated equivalent of **Dye-X**^**•+**^ (**Dye-F**^**•+**^) was therefore included in our previous study to control for these other interactions^37^. Without polarizable halogen substituents, **Dye-F**^**•+**^ is not expected to engage in halogen bonding, but background signals arising from other Cl^–^ interactions will still occur to approximately the same degree as in **Dye-Br**^**•+**^···Cl^–^ and **Dye-I**^**•+**^···Cl^–^. In the **Dye-F**^**•+**^···Cl^–^ XAS spectrum, only the edge peak between 2822 and 2828 eV and a small pre-edge signal at ~2819 eV were observed. This small pre-edge feature was also observed in the spectrum of the blank TiO_2_ substrate treated with the NOBF_4_ oxidant (TiO_2_^ox^), and was therefore attributed to background TiO_2_^ox^ ← Cl^−^ transitions arising from chloride interactions with TiO_2_ defect sites^37^. Because the pre-edge peak and pre-edge shoulder observed in the **Dye-I**^**•+**^···Cl^–^/**Dye-Br**^**•+**^···Cl^−^ XAS spectra are absent in the **Dye-F**^**•+**^···Cl^–^ spectrum, we can reliably attribute these features to halogen bonding interactions (Fig. 2b, Supplementary Fig. 4).

### X-ray transition assignments

We undertook DFT simulations to define the chemical origins of the observed XAS transitions. The M06-2X functional has proven effective in describing bond lengths for halogen bonding interactions and was therefore used to generate geometry-optimized models of the **Dye-X**^**•+**^···Cl^–^ dimers^18^. These models describe halogen bond distances of 3.26 Å and 3.40 Å for **Dye-I**^**•+**^···Cl^–^ and **Dye-Br**^**•+**^···Cl^–^, respectively (Fig. 2a). As expected, optimization of the **Dye-F**^**•+**^···Cl^–^ control did not place Cl^–^ in a halogen bonding geometry, and instead positioned it 3.98 Å above the face of a phenyl ring of **Dye-F**^**•+**^, consistent with a weak π-anion interaction rather than a halogen bonding interaction (Fig. 2a). While it is expected that these π-anion interactions will have approximately the same XAS signal for all three **Dye-X**^**•+**^ compounds, we nonetheless prepared geometry-optimized models of **Dye-I**^**•+**^···Cl^–^ and **Dye-Br**^**•+**^···Cl^–^ with chloride interacting with a phenyl group similar to **Dye-F**^**•+**^···Cl^–^. These models are denoted **Dye-I**^**•+**^···Cl^–^_π-anion_ and **Dye-Br**^**•+**^···Cl^–^_π-anion_ and position the chloride anion 3.96 Å and 4.00 Å above the face of a phenyl ring, respectively.

**Fig. 3:**
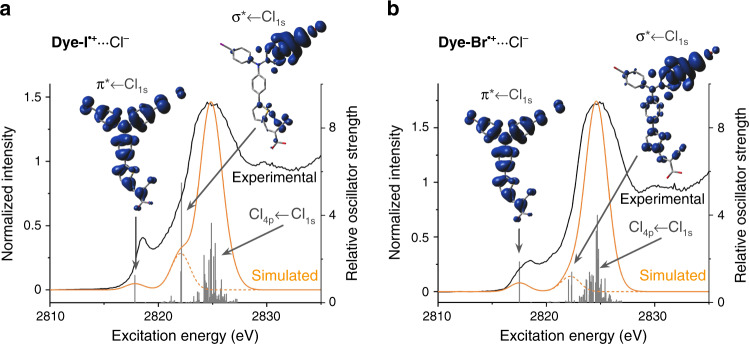
Experimental and TD-DFT simulated XAS spectra. The experimental XAS spectra (black lines) of **Dye-I**^**•+**^···Cl^–^ (**a**) and **Dye-Br**^**•+**^···Cl^–^ (**b**) and the corresponding TD-DFT simulated spectra (calculated using BP86, solid orange lines) are shown along with the relative oscillator strengths of the individual calculated transitions (vertical gray lines). The height of the simulated edge peaks were normalized to the height of the experimental edge peak. The transitions corresponding to pre-edge features were visualized as electron density difference maps (EDDMs) at an isovalue of 0.0004 (inset). The blue lobes represent positive changes in electron density corresponding to the acceptor orbital(s) of the transitions, while the donor orbital was always the chloride 1s (indicated by a negative change in electron density in red and not always visible). In order to highlight the pre-edge features resulting from *σ*- and π-covalency in **Dye-I**^**•+**^···Cl^–^ and **Dye-Br**^**•+**^···Cl^–^, truncated versions of the simulated spectra excluding the high energy Cl_4p_ ← Cl_1s_ edge peak transitions were also generated (dotted orange lines).

Time-dependant DFT (TD-DFT) simulations of the XAS spectra were performed by calculating transitions originating from the chloride 1s orbitals of the geometry-optimized models. Several DFT functionals with varying amounts of exact Hartree-Fock exchange were employed for these calculations as described in the Supplementary Methods section. Three distinct groups of transitions are predicted on the basis of the TD-DFT results for **Dye-I**^**•+**^···Cl^–^ and **Dye-Br**^**•+**^···Cl^–^ (Supplementary Figs. 5 and 6, respectively). These three groupings are easily visualized with the TD-DFT results calculated with the pure generalized gradient approximation (GGA) functional BP86 (Fig. 3): (i) a transition into the half-filled π orbital of **Dye-I**^**•+**^ or **Dye-Br**^**•+**^ with significant π-orbital contributions from Cl^–^ (π* ← Cl_1s_); (ii) multiple transitions into a σ* orbital on **Dye-I**^**•+**^ or **Dye-Br**^**•+**^ involving the 3p orbitals of Cl^–^ (σ* ← Cl_1s_); and, (iii) a cluster of many transitions from the Cl^–^ 1s orbital to an orbital with mixed Cl 4p and **Dye-I**^**•+**^ or **Dye-Br**^**•+**^ π* character.

The **Dye-F**^**•+**^···Cl^–^ spectrum was calculated to have only two groups of transitions (Supplementary Fig. 7): A weak transition from the Cl^–^ 3p orbital to the phenyl π* orbitals, consistent with a weakly covalent π-anion interaction (π* ← Cl_1s_); and the edge peak (Cl_4p_ ← Cl_1s_). These results suggest that the small pre-edge peak observed for **Dye-F**^**•+**^···Cl^–^ may arise from π-anion interactions, and not just Cl^–^ interactions with the TiO_2_ substrate as we previously assumed^37^. Simulated XAS spectra of the analogous **Dye-I**^**•+**^···Cl^–^_π-anion_ and **Dye-Br**^**•+**^···Cl^–^_π-anion_ structures shows only nominal differences in the XAS signal compared to **Dye-F**^**•+**^···Cl^–^ (Supplemental Fig. 8). Considering that these π-anion interactions would be present to the same or lesser extent for the more polarizable halogen substituents compared to the **Dye-F**^**•+**^···Cl^–^ control, they do not impact our conclusions.

TD-DFT methods are known to significantly underestimate the energy of XAS transitions as a result of the omission of occupied orbital relaxation in response to the core hole, the omission of relativistic stabilization, and other errors associated with the functional^47–49^. To correct for these errors, the energies of the XAS spectra were shifted by a fixed value until the calculated Cl_4p_ ← Cl_1s_ edge peak maximum matched the experimental edge peak maximum as described in the Supplementary Methods section. After correction, the calculated energies of the groups of transitions allow us to assign the pre-edge spectral features observed in the experimental **Dye-I**^**•+**^···Cl^–^ and **Dye-Br**^**•+**^···Cl^–^ XAS spectra. The calculated π* ← Cl_1s_ transitions appeared between 2817.4 and 2819.9 eV for **Dye-I**^**•+**^···Cl^–^ and between 2817.0 and 2819.7 eV for **Dye-Br**^**•+**^···Cl^–^ (Table 1). The well-resolved pre-edge peaks in the experimental **Dye-I**^**•+**^···Cl^–^ and **Dye-Br**^**•+**^···Cl^–^ XAS spectra were observed at 2818.6 and 2818.4 eV, respectively, in good agreement with the calculated π* ← Cl_1s_ transitions. The σ* ← Cl_1s_ transitions were calculated to appear between 2822.0 and 2824.1 eV for **Dye-I**^**•+**^···Cl^–^ and between 2822.1 and 2824.4 eV for **Dye-Br**^**•+**^···Cl^–^ (Table 1). In even the most generous case, these transitions are too high in energy by several eV to account for the well-resolved pre-edge peak, and in many cases are unresolved from the edge peak (Supplementary Figs. 5 and 6). Instead, the σ* ← Cl_1s_ transitions are in better agreement with the pre-edge shoulder observed in the 2820–2 eV range for both **Dye-I**^**•+**^···Cl^–^ and **Dye-Br**^**•+**^···Cl^–^.

**Table 1:** Calculated energies of the major pre-edge transitions for Dye-I^•+^···Cl^–^ and Dye-Br^•+^···Cl^–^.

Functional	Dye-I^•+^···Cl^–^ π* ← Cl_1s_ transition energy (eV)^a^	Dye-I^•+^···Cl^–^ σ* ← Cl_1s_ transition energy (eV)^a^	Dye-Br^•+^···Cl^–^ π* ← Cl_1s_ transition energy (eV)^a^	Dye-Br^•+^···Cl^–^ σ* ← Cl_1s_ transition energy (eV)^a^
BP86	2817.83	2822.13	2817.52	2822.34
M06-L	2817.38	2821.97	2816.97	2822.06
O3LYP	2818.56	2822.8	2818.2	2823.15
B3LYP	2818.83	2823.13	2818.53	2823.38
PBE0	2818.85	2823.12	2818.54	2823.41
M06	2819.6	2823.71	2819.27	2823.59
BH&HLYP	2819.86	2823.92	2819.61	2824.39
M06-2X	2819.9	2824.1	2819.66	2824.37

^a^Transition energies were shifted by a static correction factor as described in the Supplementary Methods section.

### π-orbital covalency

**Fig. 4:**
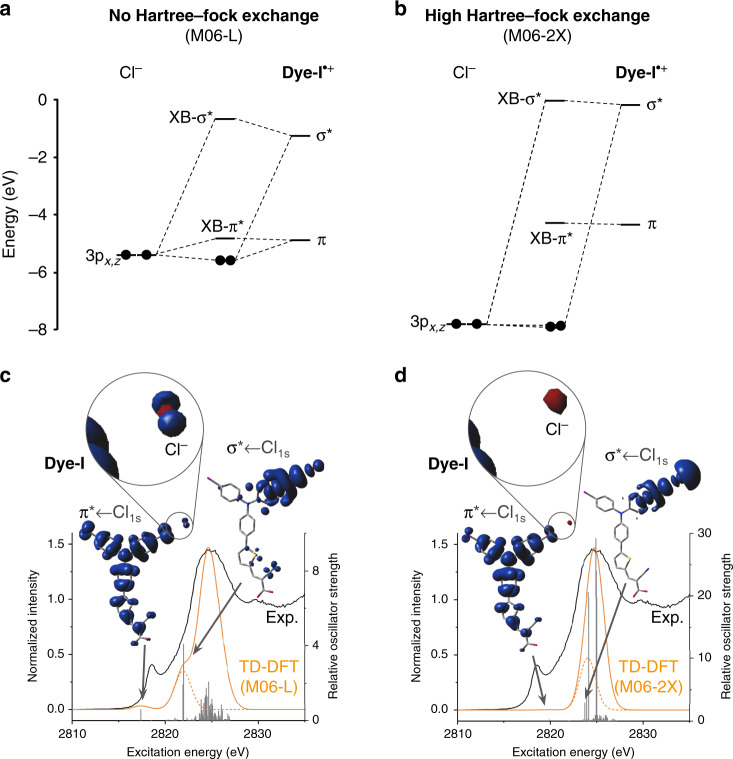
DFT functional dependence of computational results with **Dye-I**^**•+**^···Cl^–^. **a**, **b** Molecular orbital diagrams describing the orbital mixing involved in the halogen bonding interaction between Cl^–^ and **Dye-I**^**•+**^. These molecular orbital diagrams were generated from the β-spin single-electron Kohn-Sham orbital eigenvalues calculated using either the pure meta-GGA functional M06-L (**a**) or the high Hartree-Fock exchange hybrid functional M06-2X(**b**) and are simplified to consider only the orbital interactions resulting in the acceptor orbitals for the XAS pre-edge transitions. **c**, **d** TD-DFT simulated XAS spectra (orange lines) calculated using M06-L (**c**) or M06-2X(**d**) overlaid on the experimental **Dye-I**^**•+**^···Cl^–^ spectrum (black lines). The indicated pre-edge transitions are visualized with EDDMs as described in the Fig. 3 caption. In order to highlight the pre-edge features resulting from σ- and π-covalency in **Dye-I**^**•+**^···Cl^–^, truncated versions of the simulated spectrum excluding the high energy Cl_4p_ ← Cl_1s_ edge peak transitions were also generated (dotted orange line).

As discussed above, the integrated intensity of a pre-edge peak in K-edge XAS spectroscopy is directly proportional to the degree of orbital mixing that gives rise to the peak^39,46^. Bearing this in mind, the relative intensity of the observed π* ← Cl_1s_ pre-edge features compared to the edge peaks in the experimental **Dye-I**^**•+**^···Cl^–^ and **Dye-Br**^**•+**^···Cl^–^ XAS spectra suggests a significant amount of π-orbital covalency. The extent to which these pre-edge peak intensities—and by extension the π-orbital covalency—were reflected in the TD-DFT calculated XAS spectra was found to be very sensitive to the choice of functional (Fig. 4, Supplementary Figs. 5 and 6). Only the pure density functionals BP86 and M06-L and the low-HF exchange functional O3LYP predicted the π* ← Cl_1s_ pre-edge features to have any appreciable intensity compared to the edge peak. This result is not surprising because pure GGA and meta-GGA functionals are known to favor higher covalency in molecular systems^46,50^, and the BP86 functional in particular has been shown to better describe metal–ligand covalency in XAS spectra compared to hybrid functionals^51^. Electron density difference map (EDDM) plots of the π* ← Cl_1s_ transition with both **Dye-I**^**•+**^···Cl^–^ and **Dye-Br**^**•+**^···Cl^–^ reveal that mixing of the Cl 3p orbitals into the π-orbitals of the dye decreases with the intensity of the pre-edge feature. The lowest energy empty orbital of the dimers is the single-electron β-spin LUMO which can be viewed as the empty half of the π-symmetric SOMO. The trend in π* ← Cl_1s_ intensities is mirrored by the Cl^–^ involvement in the β-LUMO (Supplementary Figs. 9 and 10, Supplementary Tables 1 and 2).

**Fig. 5:**
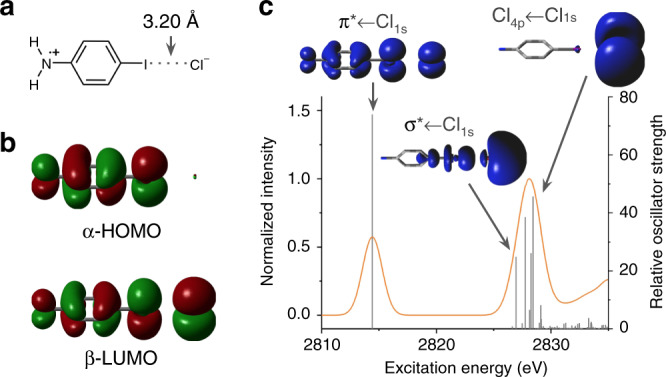
Simulated [p-iodoaniline]^•+^···Cl^–^ model system. **a** The halogen bonding adduct structure of [p-iodoaniline]^•+^···Cl^–^. **b** The α- and β-spin single-electron orbital components of the [p-iodoaniline]^•+^···Cl^–^ SOMO, the α-HOMO and β-LUMO, calculated at the OOMP2 level and plotted at an isovalue of 0.002. **c** The configuration interaction singles with MP2 doubles correction (CIS(D)) simulated XAS spectrum for [p-iodoaniline]^•+^···Cl^–^ (calculated using OOMP2, orange line) is shown along with the relative oscillator strengths of the individual calculated transitions (vertical gray lines). The height of the simulated edge peaks was normalized to 1. The transitions corresponding to pre-edge and edge peak features were visualized as EDDMs at an isovalue of 0.0008 (inset). The blue lobes represent positive changes in electron density corresponding to the acceptor orbital(s) of the transitions, while the donor orbital was always the chloride 1s (indicated by a negative change in electron density in red and not always visible).

While TD-DFT is generally the method of choice for accurately simulating K-edge XAS spectra^47–49,51^, the qualitative results are only as reliable as the underlying ground-state Kohn-Sham wavefunction. In order to verify the qualitative correctness of our TD-DFT models, we sought to examine the source of the wide discrepancy in predicted π-covalency from different DFT functionals. One possible source of this discrepancy is the self-interaction error (SIE) inherent to DFT methods (caused by the unphysical repulsion of an electron by its own density) which is known to speciously favor electron delocalization^52–57^. Wavefunction methods based on Hartree-Fock theory do not suffer from SIE and can potentially provide more qualitatively reliable results, provided that electron correlation is accounted for in a sufficiently rigorous way. While open-shell systems are persistently a challenge for wavefunction methods, Neese et al.^58^ recently demonstrated an orbital optimized Møller–Plesset 2nd order perturbation (OOMP2) method that accurately reproduces a wide range of experimental results for organic radicals^59^. We therefore employed p-iodoaniline as a truncated model system to validate our results using this method (Fig. 5). This choice of model system was supported by the fact that both the TD-DFT XAS simulations and DFT calculated frontier MOs for the [p-iodoaniline]^•+^···Cl^–^ system show the same qualitative features and functional dependence as the full **Dye-I**^**•+**^···Cl^–^ system (Supplementary Figs. 11 and 12). A comparable XAS simulation based on the OOMP2 wavefuntion was generated using the configuration interaction singles with MP2 doubles correction (CIS(D)) method. In this OOMP2-CIS(D) simulation, the π* ← Cl_1s_ pre-edge feature is still observed in the [p-iodoaniline]^•+^···Cl^–^ XAS spectrum, albeit at substantially lower energy and higher intensity relative to the TD-DFT results, and the frontier MOs reveal significant Cl^–^ involvement in the β-LUMO (Fig. 5), in qualitative agreement with the DFT results. We can therefore conclude that π-covalency in the [p-iodoaniline]^•+^···Cl^–^ system—and by extension the **Dye-X**^**•+**^···Cl^–^ systems—is not a result of SIE.

Closer examination of the **Dye-X**^**•+**^···Cl^–^ electronic structures instead points to the large disparity in predicted π-covalency being a result of differences in the donor-acceptor orbital energy gap. Zhang and Musgrave have demonstrated that the calculated HOMO-LUMO gap of molecules is highly dependant on the choice of DFT functional, and according to their study hybrid functionals consistently overestimate this gap^60^. A similar effect is observed with our **Dye-X**^**•+**^···Cl^–^ systems, as illustrated by the quantitative MO diagrams of the **Dye-I**^**•+**^···Cl^–^ halogen bonding interaction constructed using each of the tested functionals (Supplementary Figs. 13–20). The orbital energy effect is exemplified by the Minnesota functionals (Fig. 4) where the energy of the empty π-symmetry **Dye-I**^**•+**^ acceptor orbitals (*A*_π_) rises as a function of increasing exact HF exchange in the functional. In contrast, the filled 3p orbitals of the chloride nucleophile exhibit an inverse energy trend with respect to HF exchange character. As a result, the energy gap between the 3p donor orbitals on Cl^–^ and the *A*_π_ orbital on **Dye-I**^**•+**^ is increased from 0.21 eV for the GGA functional BP86 at one extreme to 3.89 eV for the hybrid BH&HLYP at the other extreme. Basic MO theory states that the degree of orbital mixing between two parent orbitals to form an MO is a function of the energy gap between the parent orbitals^61^. On this basis, the differences in π-orbital covalency predicted by the various functionals under investigation are attributed entirely to the differences in the orbital energy gap.

## Discussion

The existing model of covalency in halogen bonding only considers σ-symmetry donation of electron density from the nucleophile into the σ* orbital of the halogen bond donor^23,24,41,42^. Our study demonstrates that the observed pre-edge features in the experimental XAS spectra for **Dye-I**^**•+**^···Cl^–^ and **Dye-Br**^**•+**^···Cl^–^ can be attributed to a π-orbital interaction. While our computational models do predict an XAS transition corresponding to σ-covalency for both the **Dye-I**^**•+**^···Cl^–^ and **Dye-Br**^**•+**^···Cl^–^ dimers, all of the methods we employ predict this transition to be too high in energy by at least 3 eV relative to the edge peak to be resolved (Fig. 3, Supplementary Figs. 5 and 6). We instead propose that the shoulders experimentally observed in the 2820–2 eV range are consequent of the σ-interactions. This conclusion is further supported by the calculated intensities of the σ* ← Cl_1s_ transition for both **Dye-I**^**•+**^···Cl^–^ and **Dye-Br**^**•+**^···Cl^–^ mirroring the relative prominence of this shoulder feature in the experimental spectra.

The π* ← Cl_1s_ pre-edge peak at ~2818.5 eV must therefore be the result of a much lower energy orbital. For both the **Dye-I**^**•+**^ and **Dye-Br**^**•+**^ XB-donors the lowest energy empty orbital is the π-symmetry β-LUMO that is orthogonal to the halogen bond axis. The lowest energy acceptor orbitals that can possibly result from these halogen bonds are therefore mixing of the Cl^–^ 3p orbitals into this β-LUMO. Given the symmetry of the orbitals involved and the linear geometry of the halogen bond, this interaction must also have π-symmetry analogous to the schematic in Fig. 1. Only those computational methods that account for π-orbital covalency correctly predicted the pre-edge features of the **Dye-I**^**•+**^···Cl^–^ and **Dye-Br**^**•+**^···Cl^–^ experimental XAS spectra.

This study provides an expanded description of the halogen bond. The standard covalent model of the halogen bonding interaction describes σ-symmetric orbital overlap, but we show that π-symmetric overlap is also possible and can have a significant impact on the observable electronic properties of halogen bonded molecules. We demonstrate this effect using a radical cation XB-donor and it remains to be seen how generalizable it is. Nevertheless, we believe that this effect can be extended beyond open-shell systems, such as in the situation described by Supplementary Fig. 1c. In much the same way as metal coordination bonds can contain a π-covalent component, π-covalency in halogen bonds should be considered whenever the nucleophile and electrophile have empty and filled π-symmetric orbitals that are close in energy. The majority of hybrid DFT functionals commonly used to model halogen bonding significantly underestimate this π-covalency. This broadened description of the halogen bond highlights new opportunities to design XB-donors and acceptors with the ability to form intermolecular π-interactions. Halogen bonds could thereby be used to modify reactivity and tune electronic properties in ways that cannot be achieved by other intermolecular interactions.

## Supplementary information

Supplementary Information

Description of Additional Supplementary Files

Supplementary Data 1

## Data Availability

Supplementary Figures, Supplementary Methods and Supplementary Tables are included in the Supplementary Information file. DFT optimized molecular geometries are reported in the Supplementary Data 1 file. Additional data supporting these findings are available from the corresponding author upon request. Source data are provided with this paper.

## Source data

Source Data
