# Supplementary material for: π covalency in the halogen bond
Source: Nat Commun. 2020 Jul 3;11:3310. doi: 10.1038/s41467-020-17122-7 (PMC7335087; doi:10.1038/s41467-020-17122-7)
Supplement: Supplementary file 1 — Supplementary Information [file 41467_2020_17122_MOESM1_ESM.pdf]

Supplementary Information

π covalency in the halogen bond

Kellett, et al.

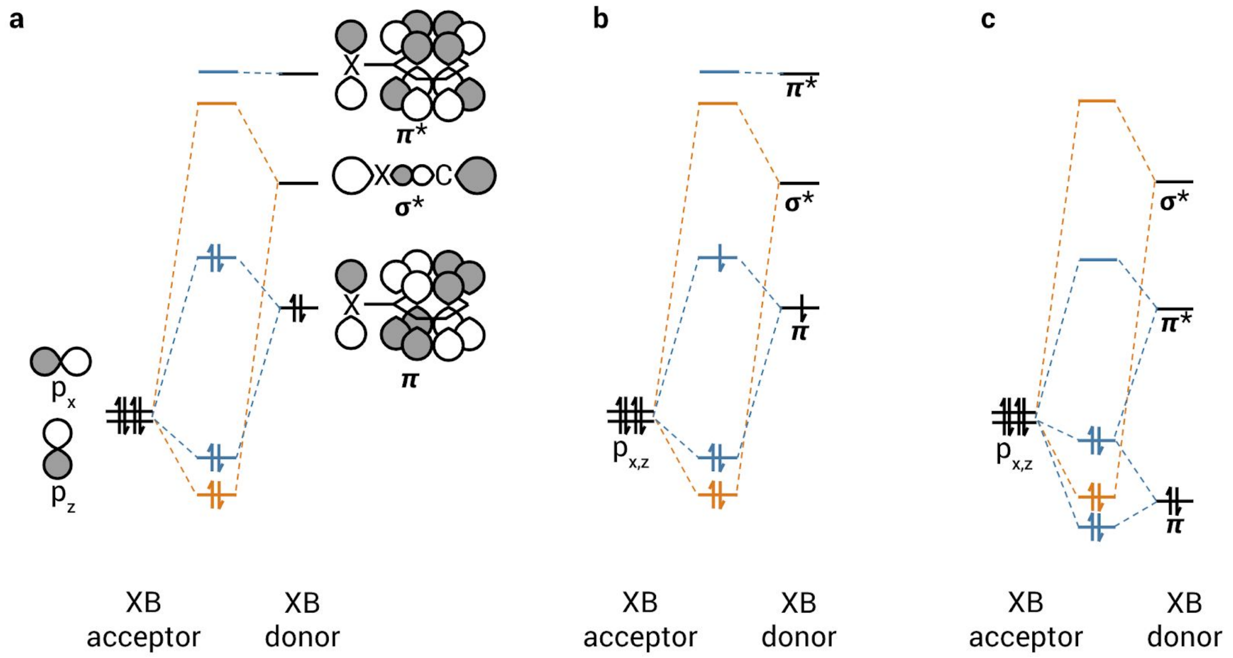

Supplementary Fig. 1 | π orbital covalency in halogen bonds. Schematic MO diagrams describing the orbital interactions involved in halogen bonding between a π-donating XB-acceptor and a conjugated XB-donor with high energy π and π* orbitals (**a**), a partially filled π orbital (**b**), or low energy π and π* orbitals (**c**). Generic illustrations of the relevant orbitals on the XB-donor and XB-acceptor are depicted in panel **a**. σ-symmetric interactions are indicated by orange lines while π-symmetric interactions are indicated by blue lines.

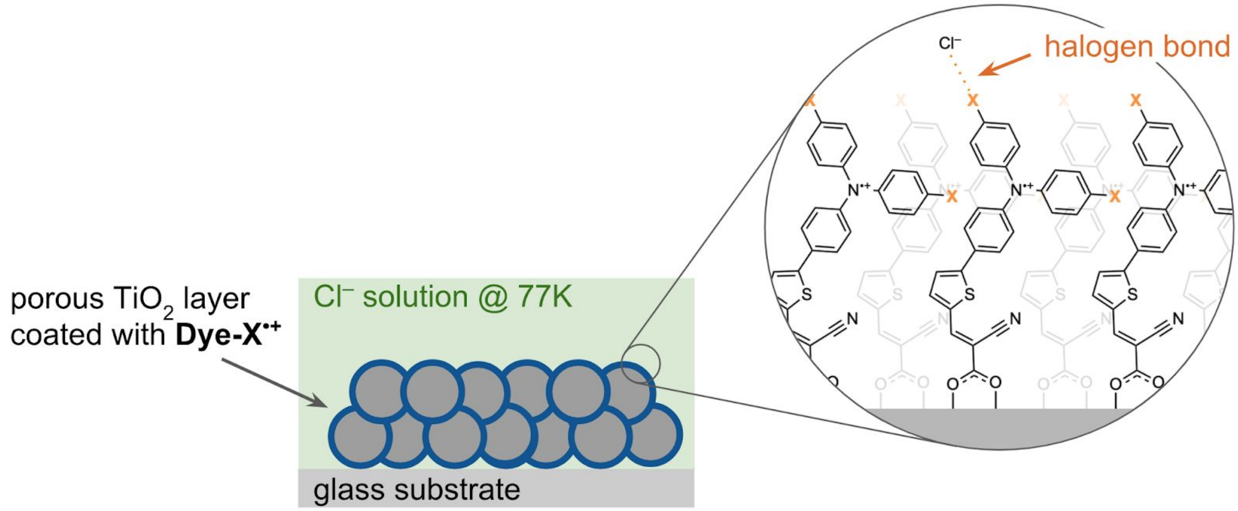

Supplementary Fig. 2 | Schematic of experimental XAS samples. This figure presents a simplified schematic representation of the sample configuration employed in our previously reported **Dye-X**$^{•+}$···Cl^- XAS studies.[1] Briefly, the neutral **Dye-X** compounds were adsorbed to a mesoporous TiO_2 layer on glass, oxidized with nitrosonium tetrafluoroborate, immersed in a 100 mM solution of tetrabutylammonium chloride in acetonitrile, and frozen at 77 K prior to data collection.

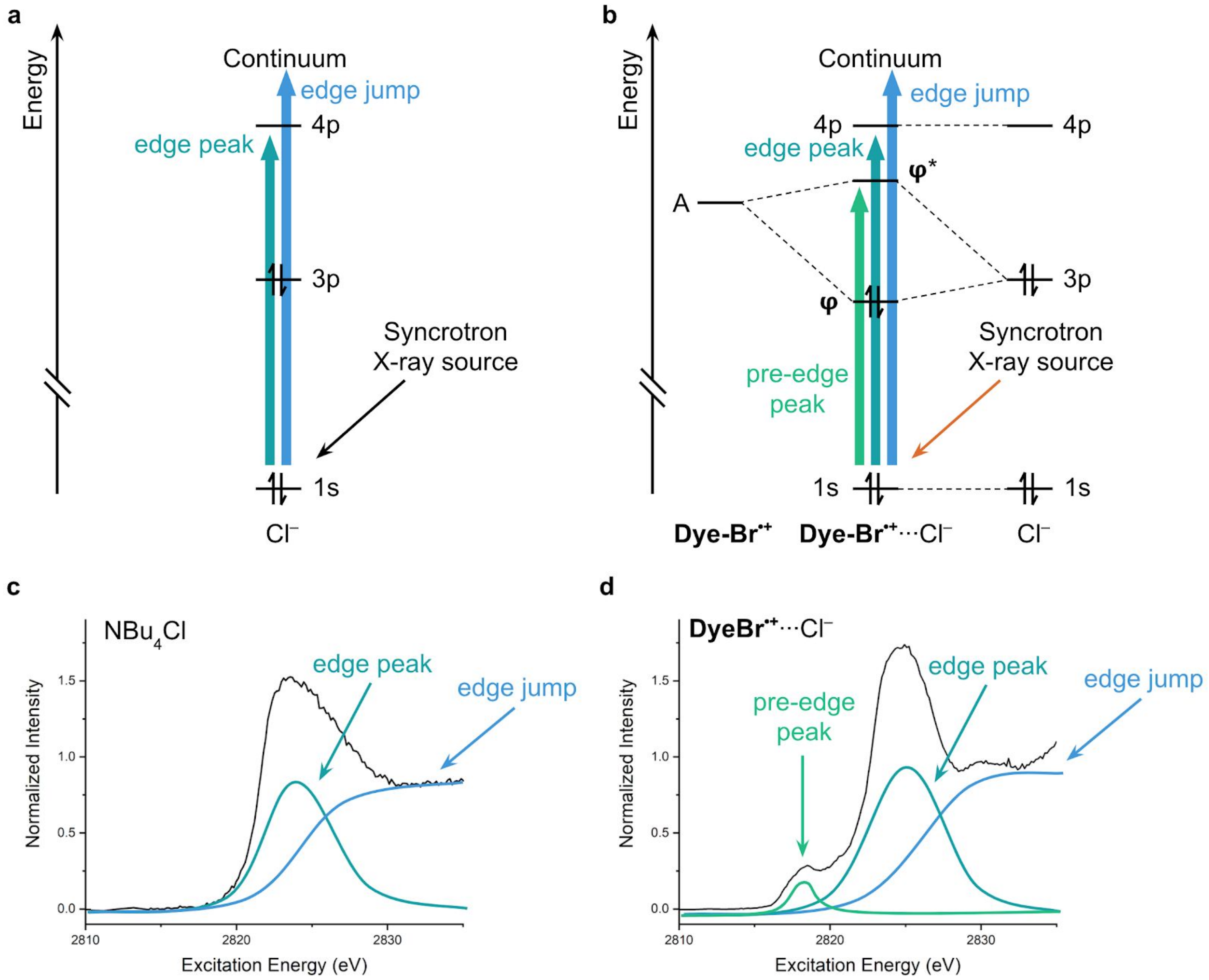

Supplementary Fig. 3 | Chlorine K-edge X-ray absorption spectroscopy (XAS) schematic. **a**, **b**, Schematic representation of the primary excitation processes at work in a pure chloride sample (**a**) and chloride in the presence of a **Dye-Br**$^{\bullet+}$ halogen bond donor (**b**). **c**, **d**, The experimental XAS spectra of the tetrabutylammonium chloride control (**c**) and the **Dye-X**$^{\bullet+}$···Cl^- sample (**d**) overlaid with approximate Gaussian curves highlighting the effect that each major transition has on the observed spectra.[1]

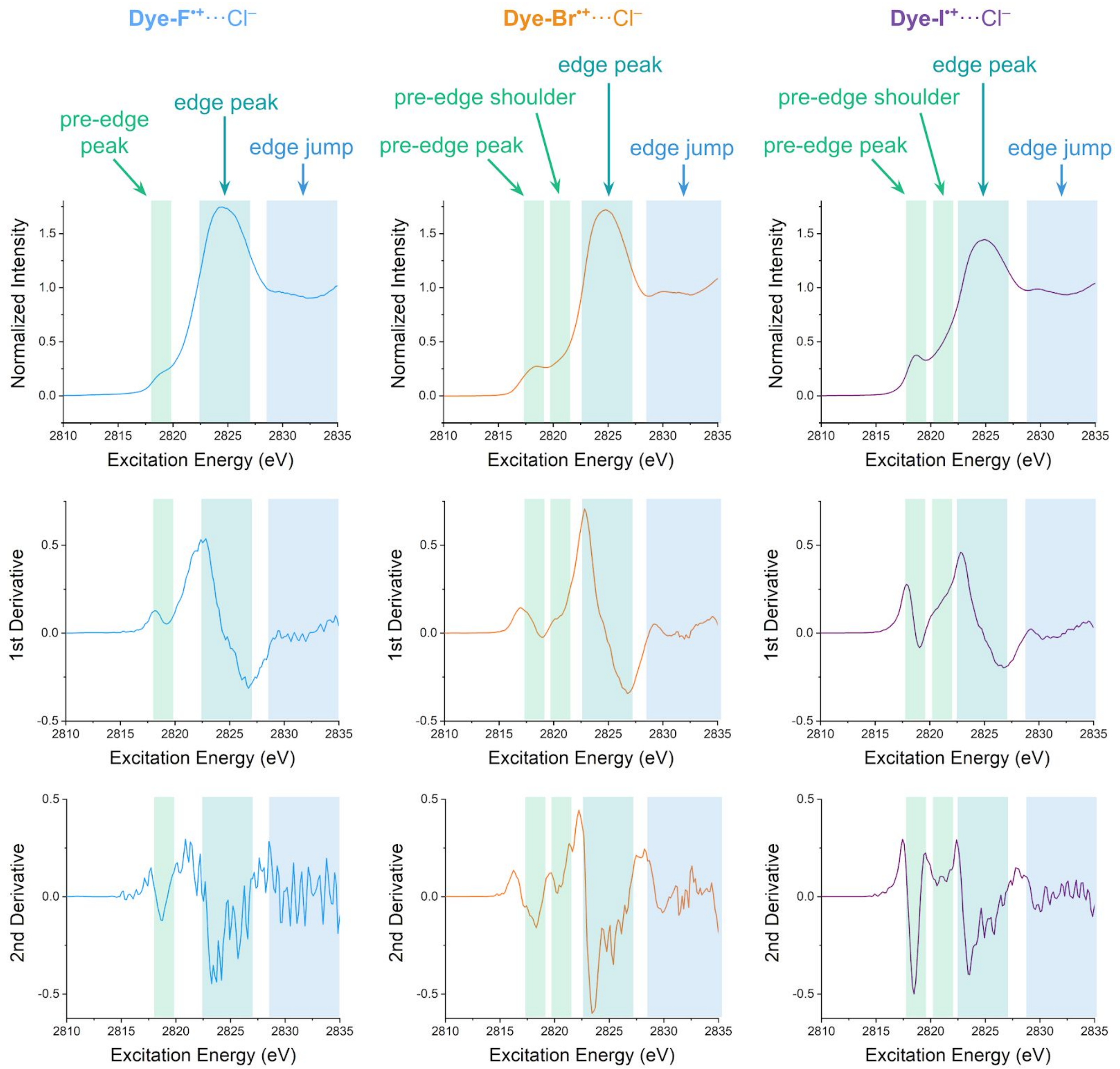

Supplementary Fig. 4 | Spectral feature assignments of Dye-X$^{•+}$⋯Cl^{-} spectra. The experimental XAS spectra of the indicated **Dye-X$^{•+}$**⋯Cl^{-} samples are plotted at the top,[1] with the first and second derivatives of the data (calculated using the central difference method) plotted on the second and third rows, respectively. For the purposes of this analysis, the experimental data was smoothed with a moving average of 7 points to reduce the noise in the 1st and 2nd derivative plots.

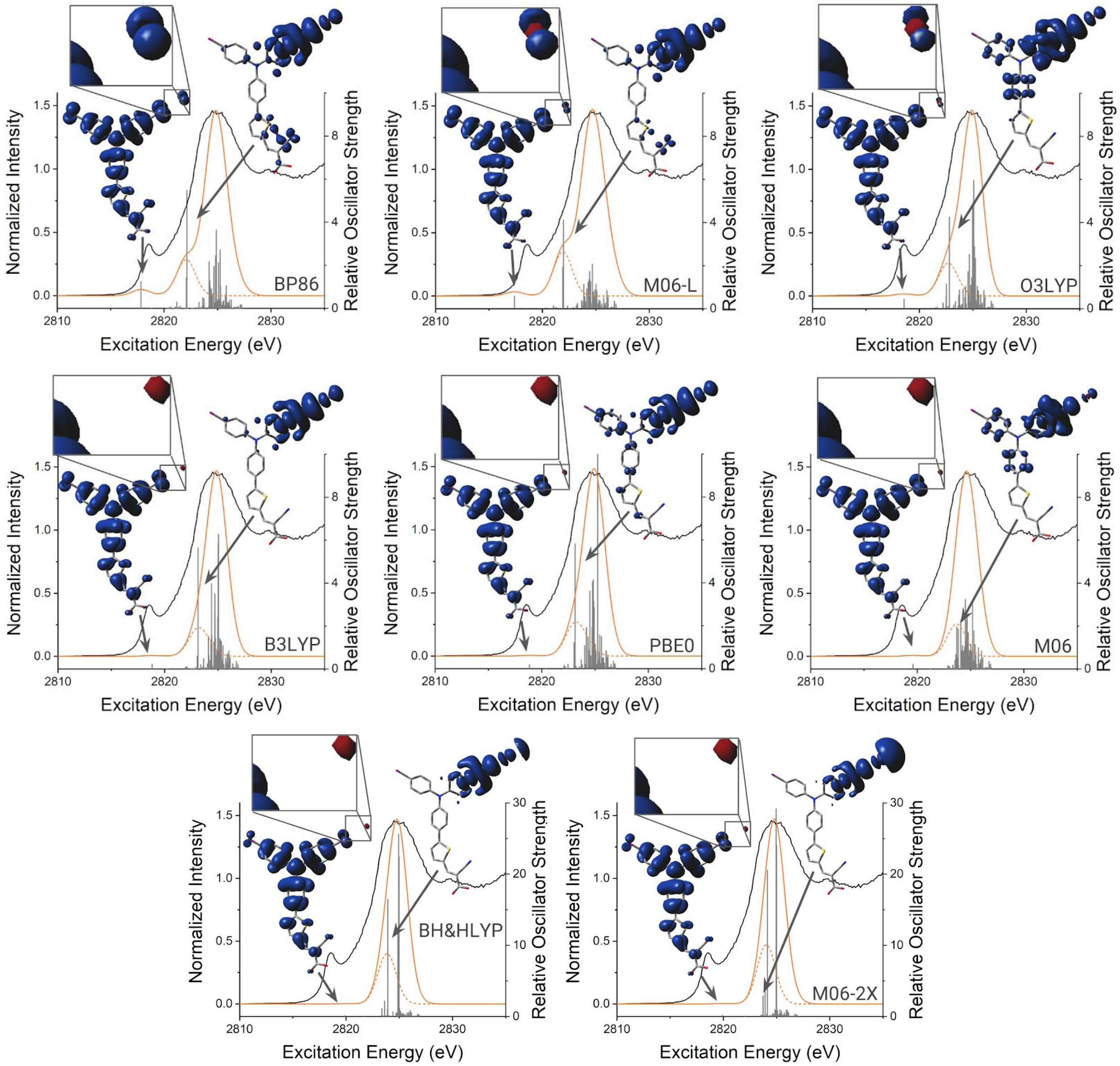

Supplementary Fig. 5 | TD-DFT simulated XAS spectra for Dye-I$^{\bullet+}\cdots$Cl^{-}. The experimental XAS spectrum (black lines) of **Dye-I**$^{\bullet+}\cdots$Cl^{-} and the corresponding TD-DFT simulated spectra (solid orange lines) calculated with the indicated DFT functionals are shown along with the relative oscillator strengths of the individual calculated transitions (vertical grey lines). The height of the simulated spectra were normalized to the height of the experimental edge peak. The transitions corresponding to pre-edge features were visualized as electron density difference maps at an isovalue of 0.0004 (inset). The blue lobes represent positive changes in electron density corresponding to the acceptor orbital(s) of the transitions, while the donor orbital was always the chloride 1s (indicated by a negative change in electron density in red and not always visible). In order to highlight the pre-edge features resulting from σ- and π-covalency in **Dye-I**$^{\bullet+}\cdots$Cl^{-}, truncated versions of the simulated spectra excluding the high energy $Cl_{4p}\leftarrow Cl_{1s}$ edge peak transitions were also generated (dotted orange lines).

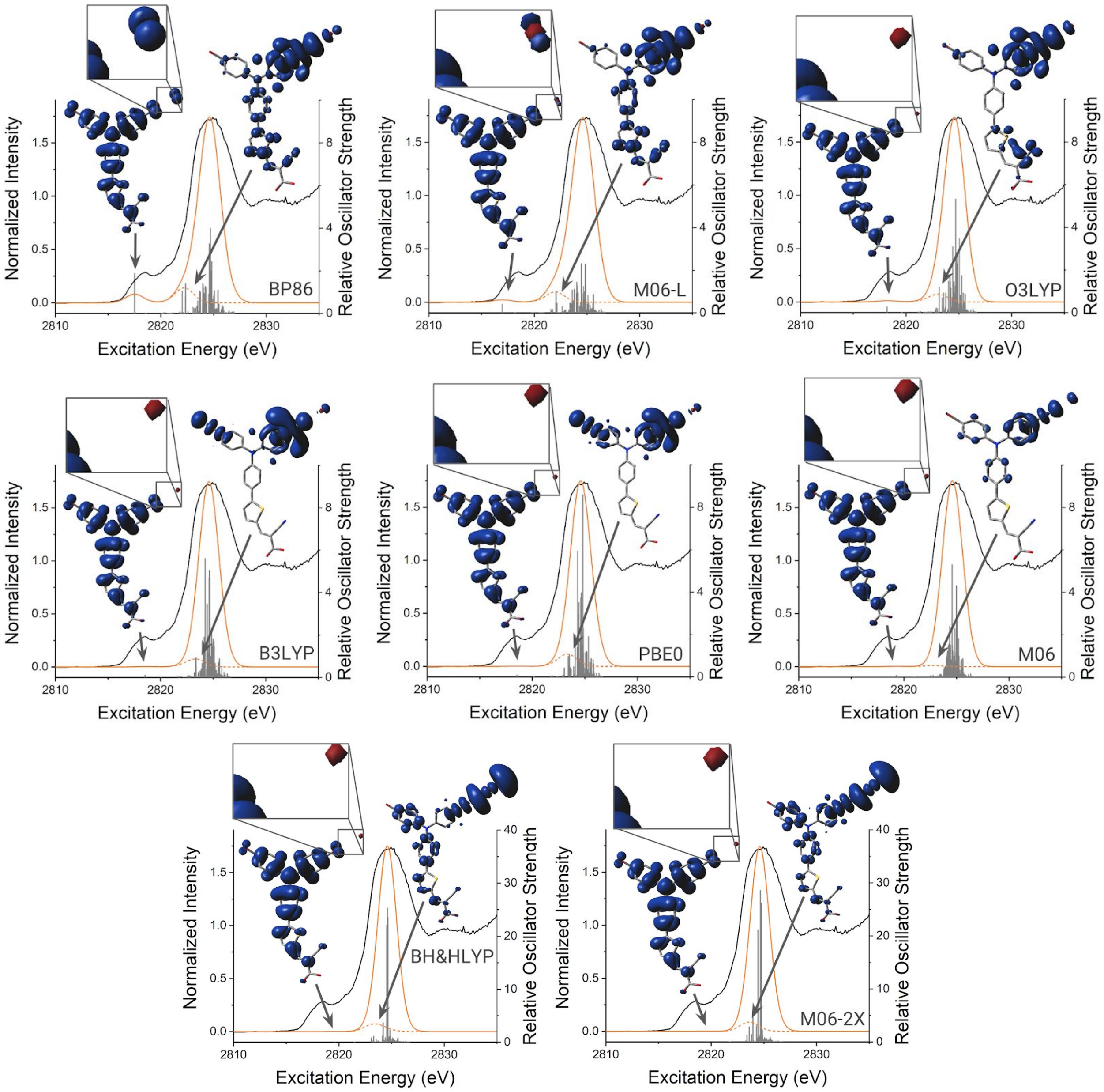

Supplementary Fig. 6 | TD-DFT simulated XAS spectra for Dye-Br$^{\bullet+}$···Cl^{-}. The experimental XAS spectrum (black lines) of **Dye-Br**$^{\bullet+}$···Cl^{-} and the corresponding TD-DFT simulated spectra (solid orange lines) calculated with the indicated DFT functionals are shown along with the relative oscillator strengths of the individual calculated transitions (vertical grey lines). The height of the simulated edge peak were normalized to the height of the experimental edge peak. The transitions corresponding to pre-edge features were visualized as electron density difference maps at an isovalue of 0.0004 (inset). The blue lobes represent positive changes in electron density corresponding to the acceptor orbital(s) of the transitions, while the donor orbital was always the chloride 1s (indicated by a negative change in electron density in red and not always visible). In order to highlight the pre-edge features resulting from σ- and π-covalency in **Dye-Br**$^{\bullet+}$···Cl^{-}, truncated versions of the simulated spectra excluding the high energy Cl_{4p}←Cl_{1s} edge peak transitions were also generated (dotted orange lines).

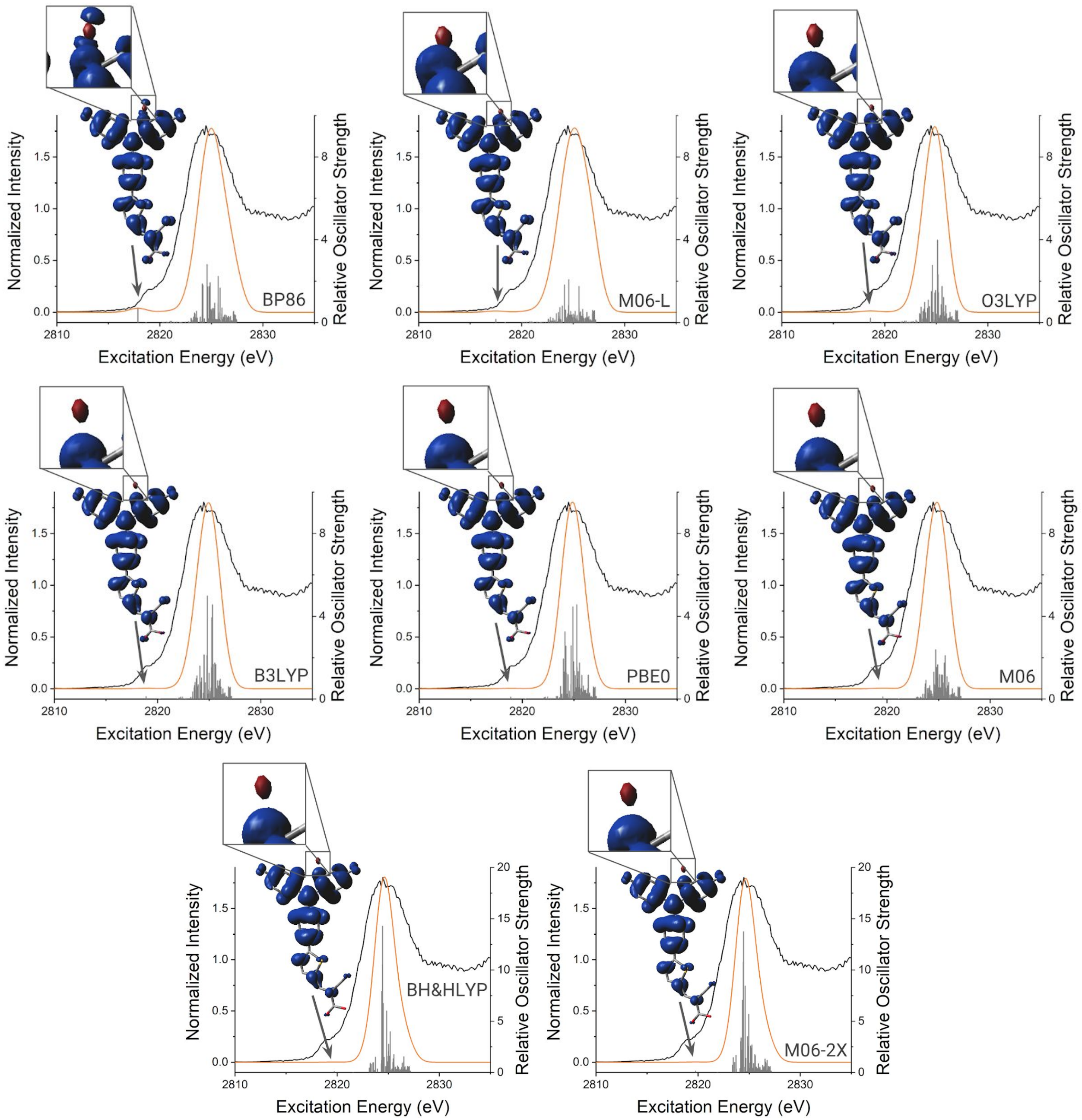

Supplementary Fig. 7 | TD-DFT simulated XAS spectra for Dye-F$^{\bullet+}\cdots$Cl^{-}. The experimental XAS spectrum (black lines) of **Dye-F$^{\bullet+}$**$\cdots$Cl^{-} and the corresponding TD-DFT simulated spectra (solid orange lines) calculated with the indicated DFT functionals are shown along with the relative oscillator strengths of the individual calculated transitions (vertical grey lines). The height of the simulated spectra were normalized to the height of the experimental edge peak. The transitions corresponding to pre-edge features were visualized as electron density difference maps at an isovalue of 0.0004 (inset). The blue lobes represent positive changes in electron density corresponding to the acceptor orbital(s) of the transitions, while the donor orbital was always the chloride 1s (indicated by a negative change in electron density in red and not always visible).

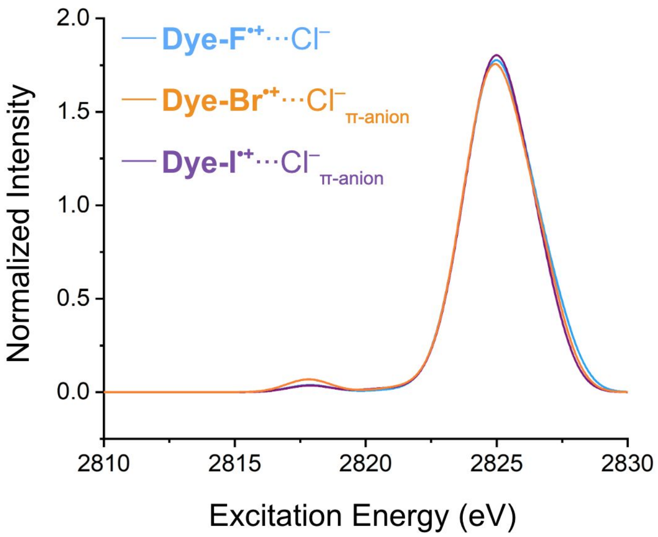

Supplementary Fig. 8 | Simulated XAS spectra of the Dye-Br$^{•+}$···Cl$^{-}_{\pi\text{-anion}}$, Dye-I$^{•+}$···Cl$^{-}_{\pi\text{-anion}}$, and Dye-F$^{•+}$···Cl^{-}. The simulated XAS spectra of the **Dye-Br$^{•+}$**···Cl$^{-}_{\pi\text{-anion}}$ structure (orange line) and the **Dye-I$^{•+}$**···Cl$^{-}_{\pi\text{-anion}}$ structure (purple line) compared to the simulated XAS spectrum of the **Dye-F$^{•+}$**···Cl^{-} control (blue line). Simulated spectra were generated using the BP86 functional.

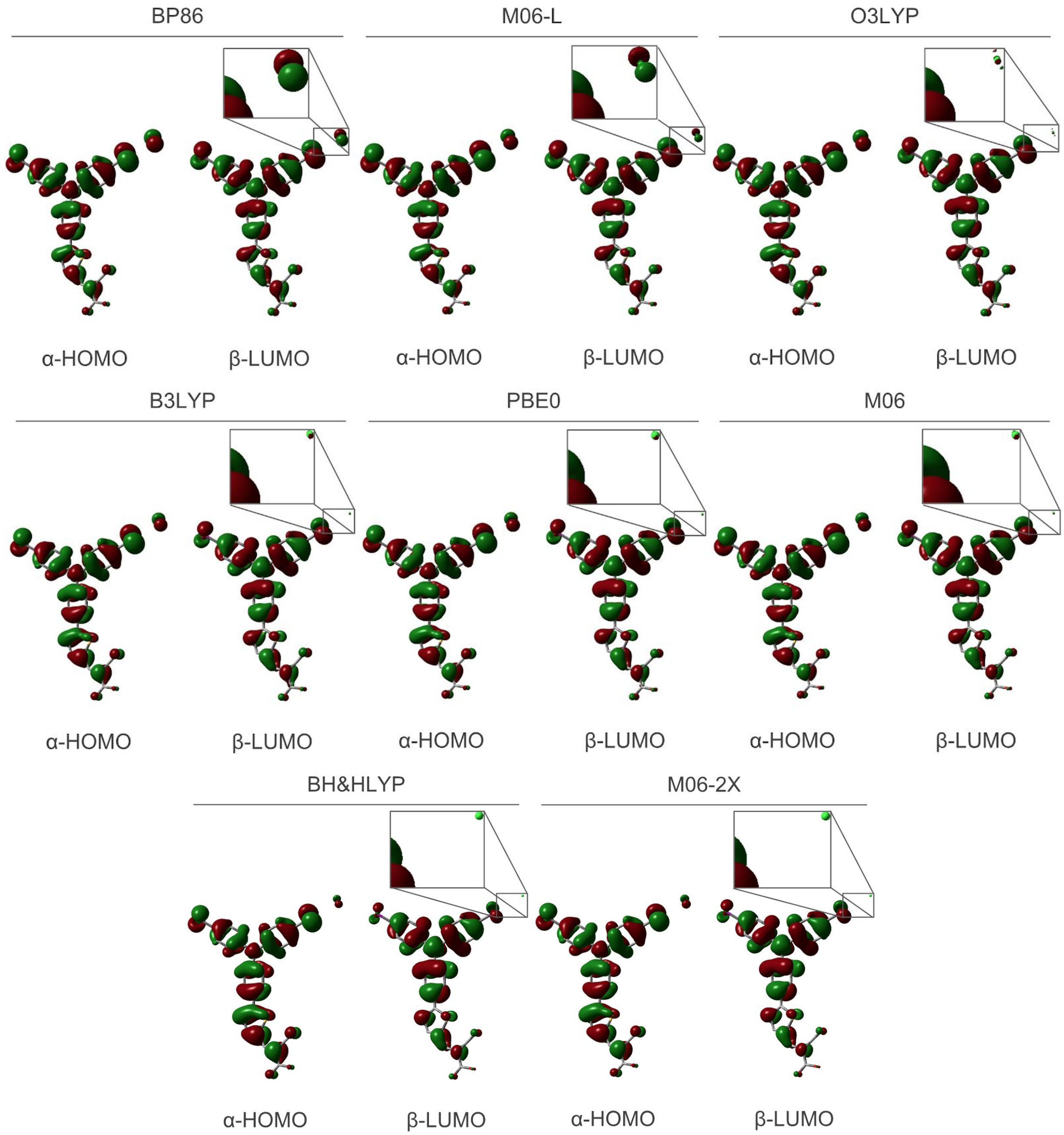

Supplementary Fig. 9 | Frontier MO plots of Dye-I$^{•+}$···Cl$^{–}$. The one-electron α and β spin Kohn-Sham orbital components of the **Dye-I$^{•+}$**···Cl$^{–}$ SOMO, the α-HOMO and β-LUMO, derived from single-point energy calculations with the indicated DFT functional are plotted at an isovalue of 0.002.

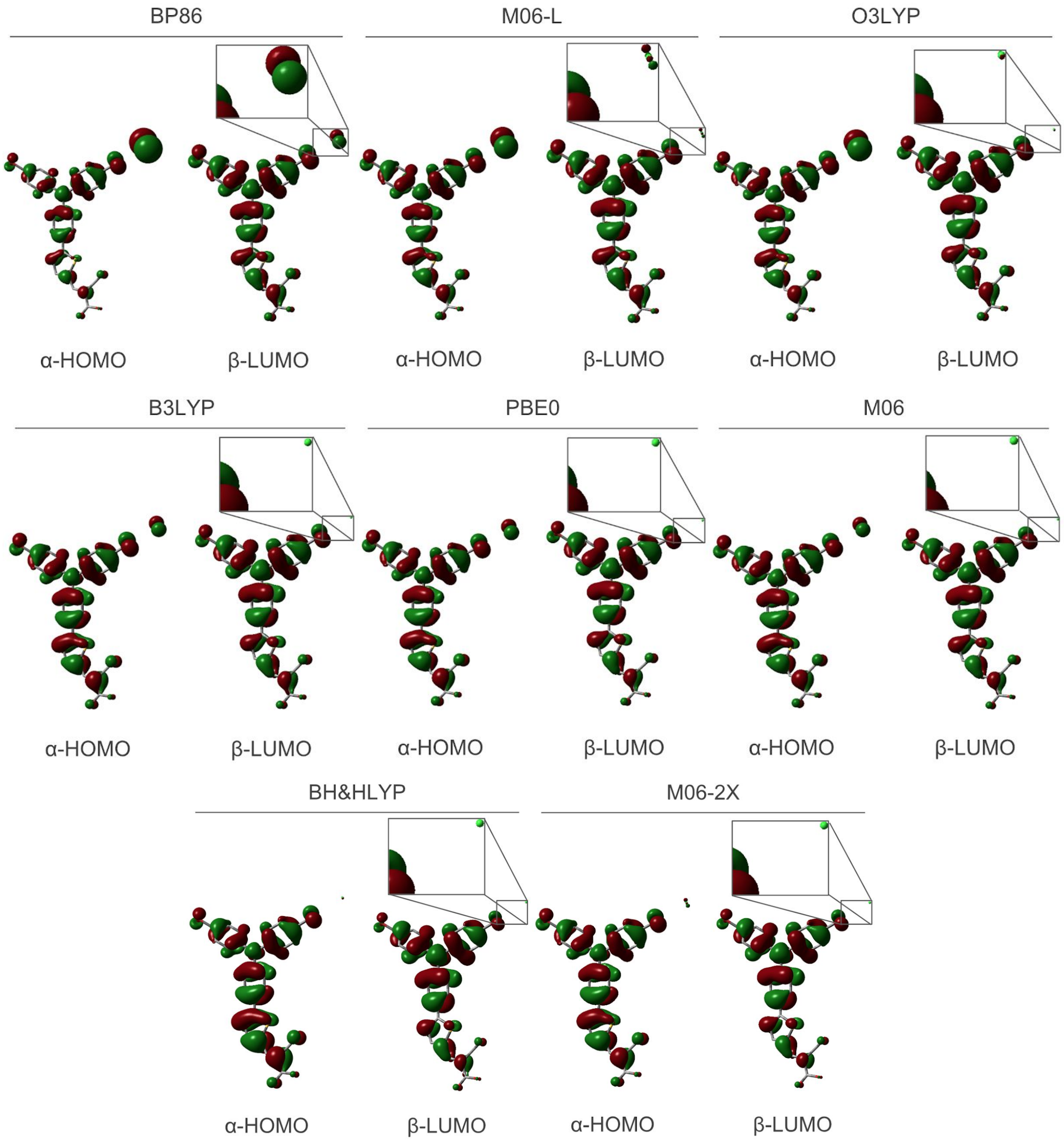

Supplementary Fig. 10 | Beta-LUMO MO plots of Dye-Br$^{\bullet+}$···Cl^{-}. The one-electron α and β spin Kohn-Sham orbital components of the **Dye-Br$^{\bullet+}$**···Cl^{-} SOMO, the α-HOMO and β-LUMO, derived from single-point energy calculations with the indicated DFT functional are plotted at an isovalue of 0.002.

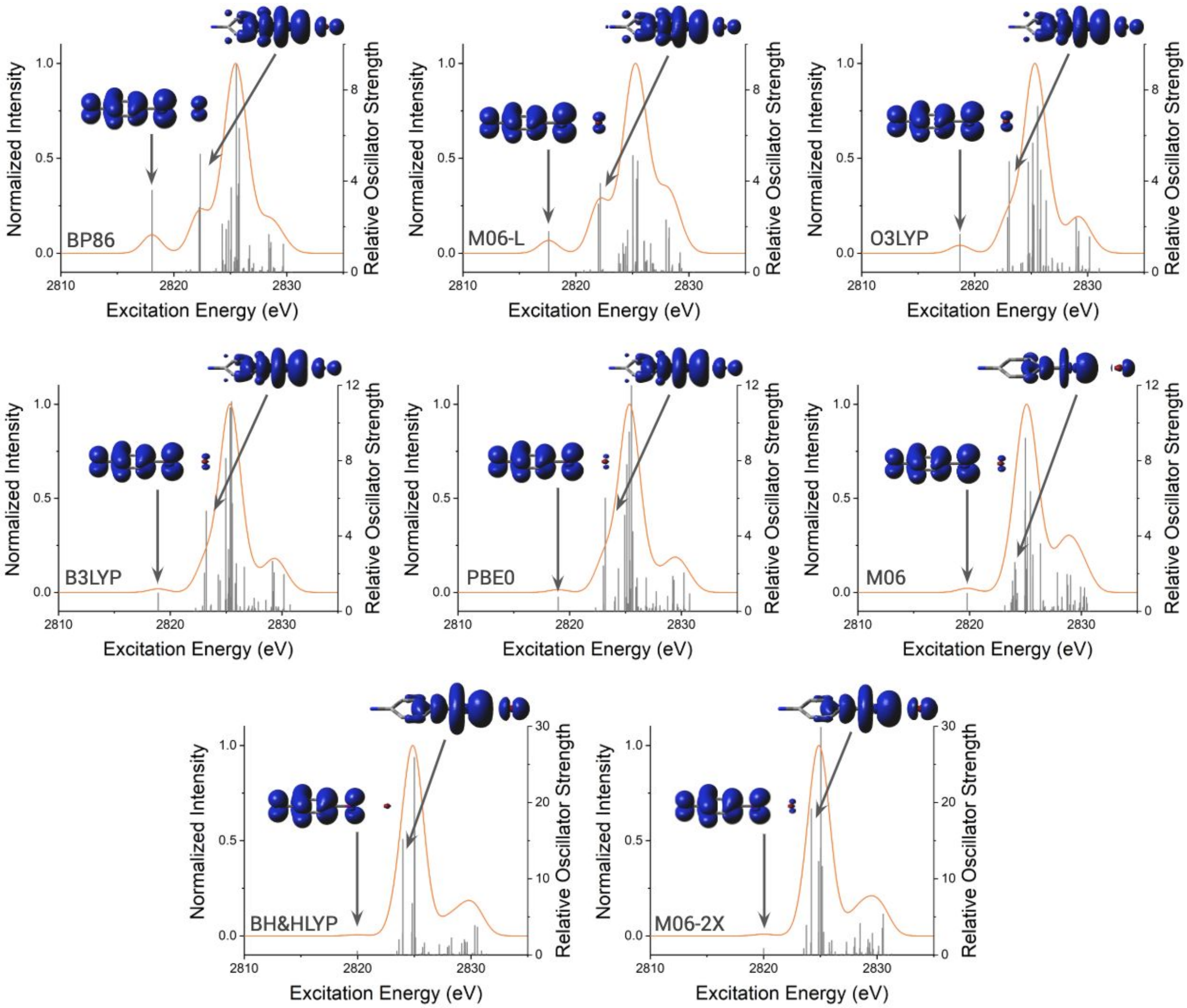

Supplementary Fig. 11 | TD-DFT simulated XAS spectra for $[\text{p-iodoaniline}]^{\bullet+}\cdots Cl^{-}$. The TD-DFT simulated XAS spectra (orange lines) of $[\text{p-iodoaniline}]^{\bullet+}\cdots Cl^{-}$ calculated with the indicated DFT functionals are shown along with the relative oscillator strengths of the individual calculated transitions (vertical grey lines). The height of the simulated edge peak were normalized to 1. The transitions corresponding to pre-edge features were visualized as electron density difference maps at an isovalue of 0.0008 (inset). The blue lobes represent positive changes in electron density corresponding to the acceptor orbital(s) of the transitions, while the donor orbital was always the chloride 1s (indicated by a negative change in electron density in red and not always visible).

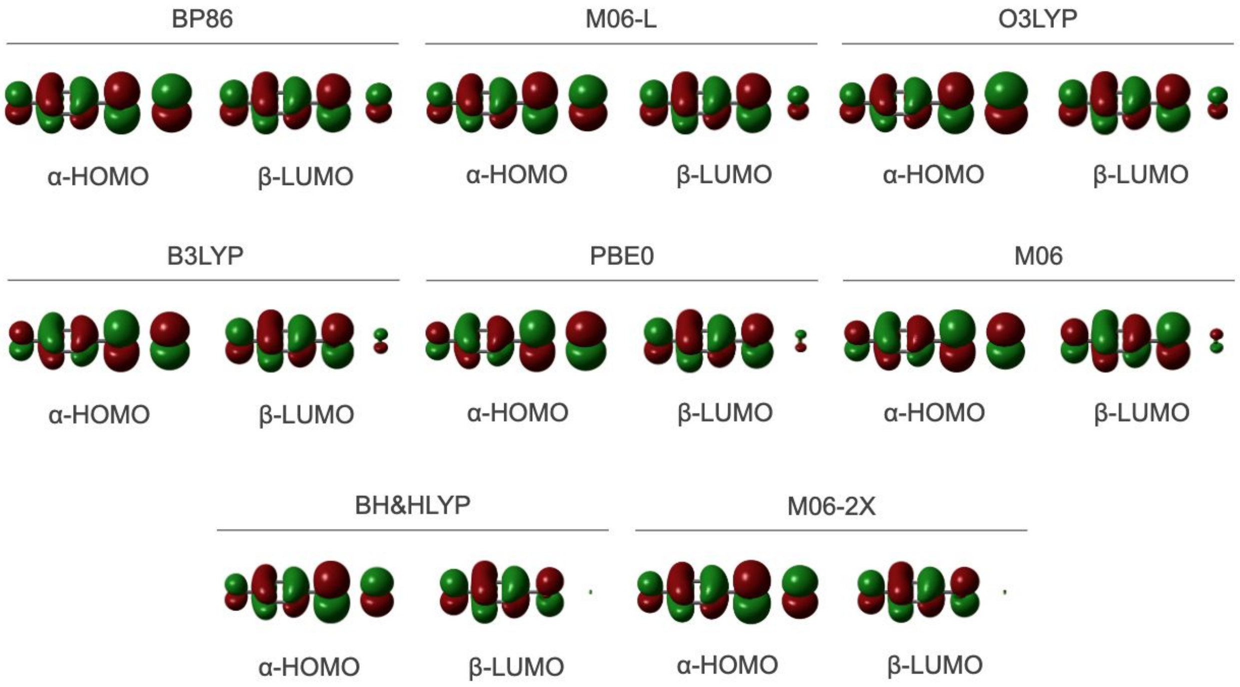

Supplementary Fig. 12 | Frontier MO plots of [p-iodoaniline]$^{\bullet+}\cdots$Cl^{-}. The one-electron α and β spin Kohn-Sham orbital components of the [p-iodoaniline]$^{\bullet+}\cdots$Cl^{-} SOMO, the α-HOMO and β-LUMO, derived from single-point energy calculations with the indicated DFT functional are plotted at an isovalue of 0.002.

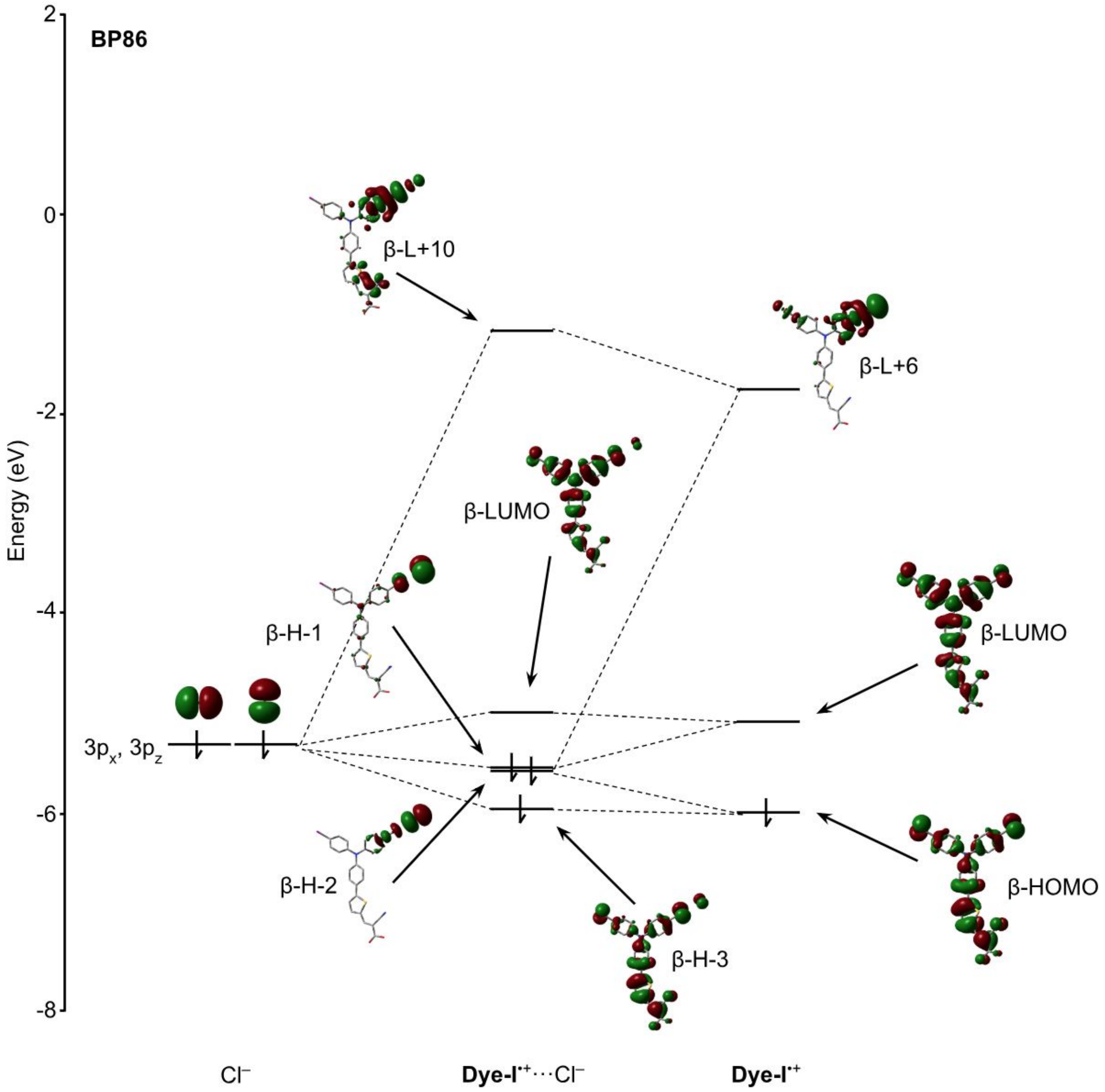

Supplementary Fig. 13 | Dye-I$^{•+}$···Cl$^{–}$ MO mixing diagram with BP86. Quantitative MO diagrams were generated of the halogen bonding interaction between **Dye-I$^{•+}$** and Cl$^{–}$. Only the β-spin single-electron Kohn-Sham orbitals were considered. The eigenvalues for each MO were calculated using BP86. These MOs were visualized at an isovalue of 0.002.

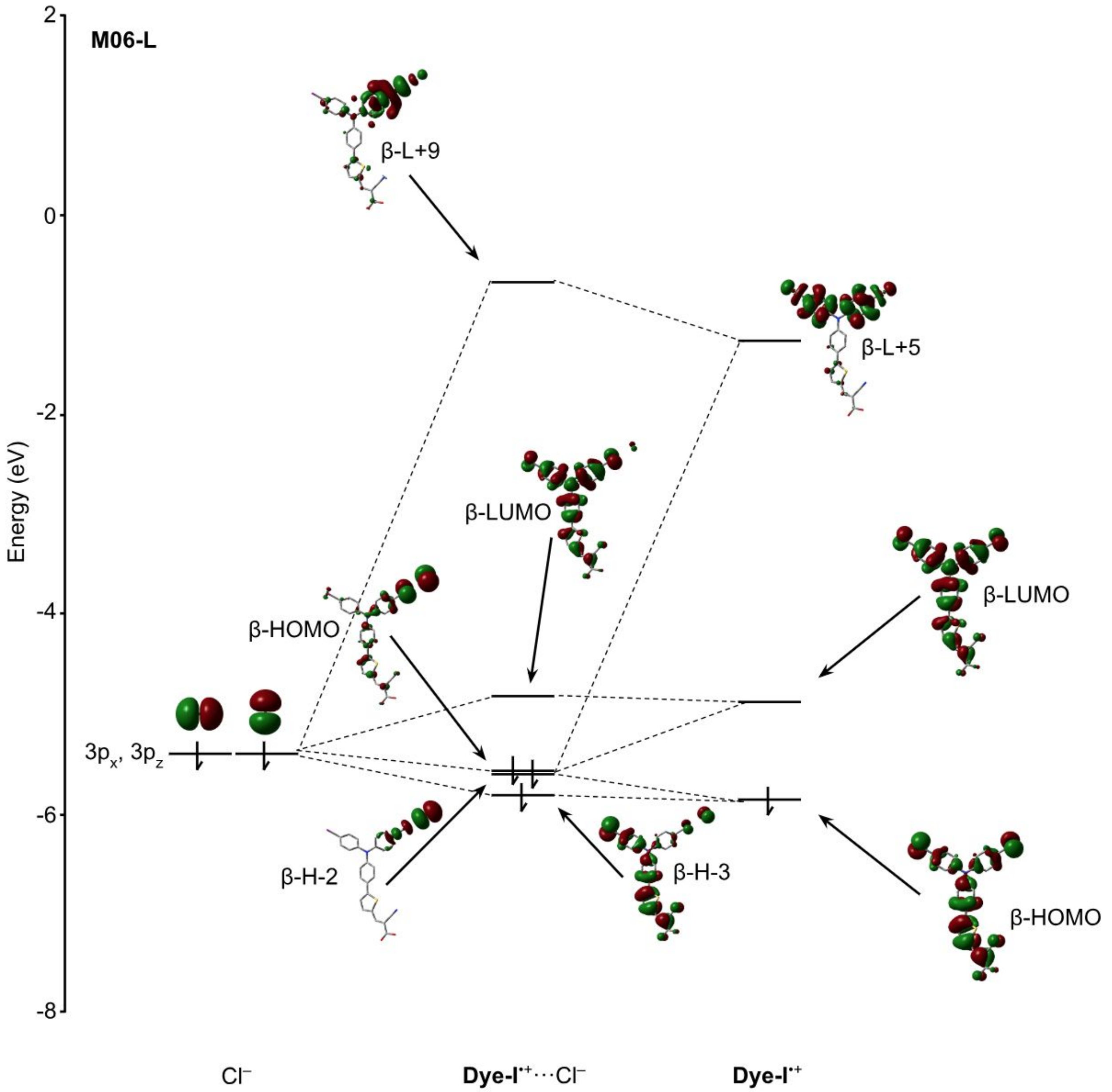

Supplementary Fig. 14 | Dye-I$^{•+}$···Cl$^-$ MO mixing diagram with M06-L. Quantitative MO diagrams were generated of the halogen bonding interaction between **Dye-I$^{•+}$** and Cl$^-$. Only the β-spin single-electron Kohn-Sham orbitals were considered. The eigenvalues for each MO were calculated using M06-L. These MOs were visualized at an isovalue of 0.002.

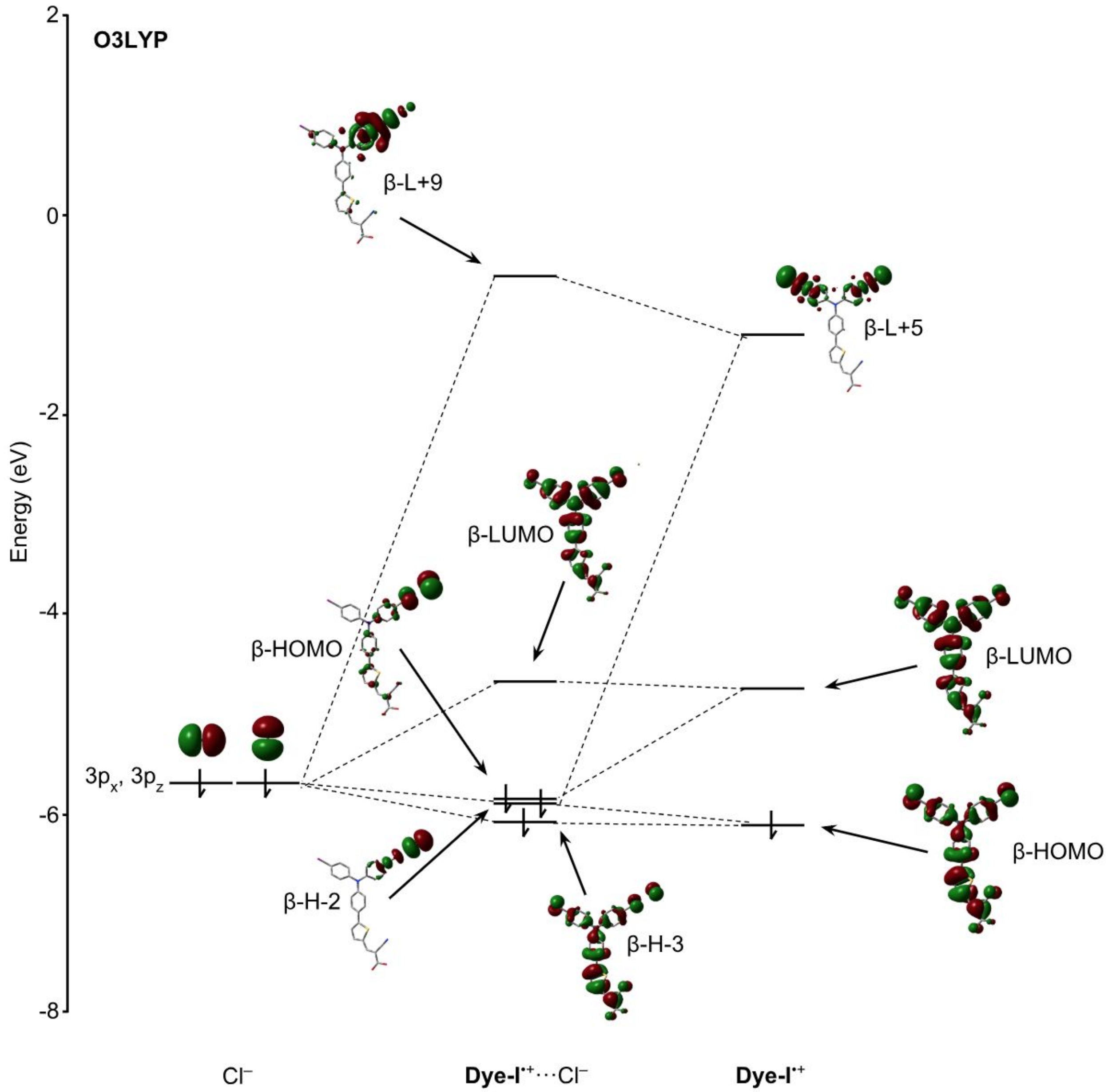

Supplementary Fig. 15 | Dye-I$^{\bullet+}$···Cl$^-$ MO mixing diagram with O3LYP. Quantitative MO diagrams were generated of the halogen bonding interaction between **Dye-I$^{\bullet+}$** and Cl$^-$. Only the β-spin single-electron Kohn-Sham orbitals were considered. The eigenvalues for each MO were calculated using O3LYP. These MOs were visualized at an isovalue of 0.002.

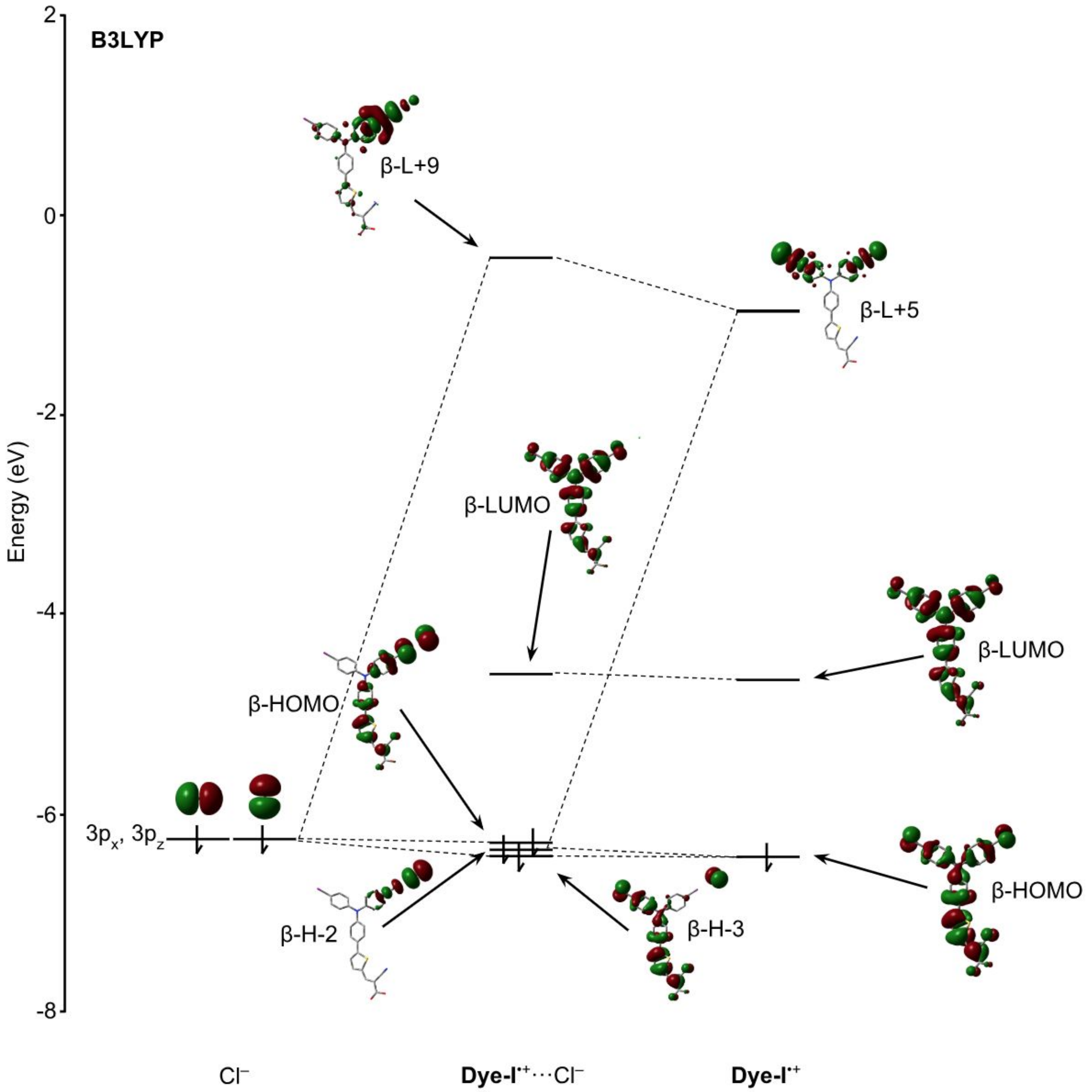

Supplementary Fig. 16 | Dye-I•+···Cl⁻ MO mixing diagram with B3LYP. Quantitative MO diagrams were generated of the halogen bonding interaction between **Dye-I•+** and Cl⁻. Only the β-spin single-electron Kohn-Sham orbitals were considered. The eigenvalues for each MO were calculated using B3LYP. These MOs were visualized at an isovalue of 0.002.

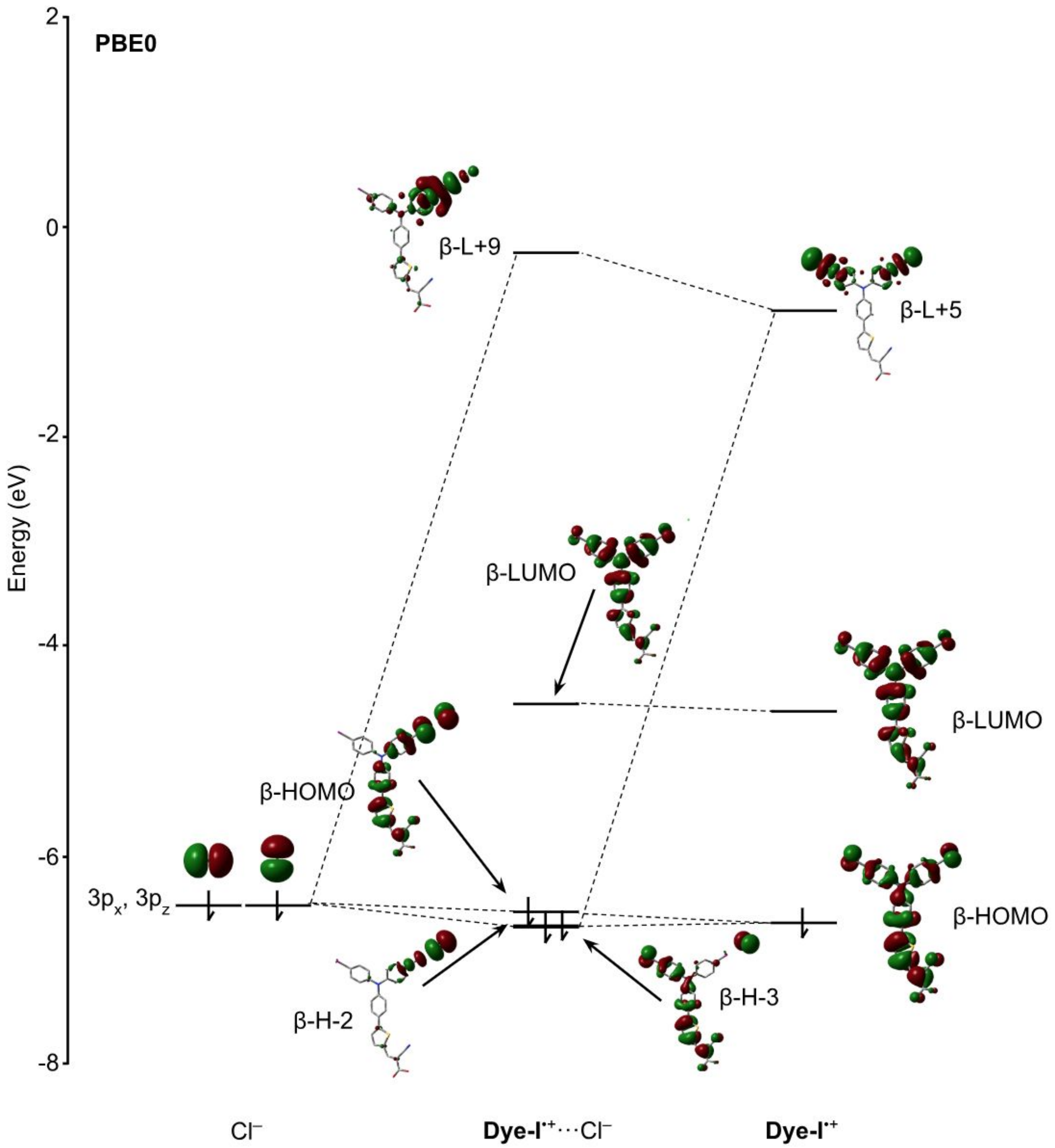

Supplementary Fig. 17 | Dye-I$^{•+}$···Cl^{-} MO mixing diagram with PBE0. Quantitative MO diagrams were generated of the halogen bonding interaction between **Dye-I$^{•+}$** and Cl^{-}. Only the β-spin single-electron Kohn-Sham orbitals were considered. The eigenvalues for each MO were calculated using PBE0. These MOs were visualized at an isovalue of 0.002.

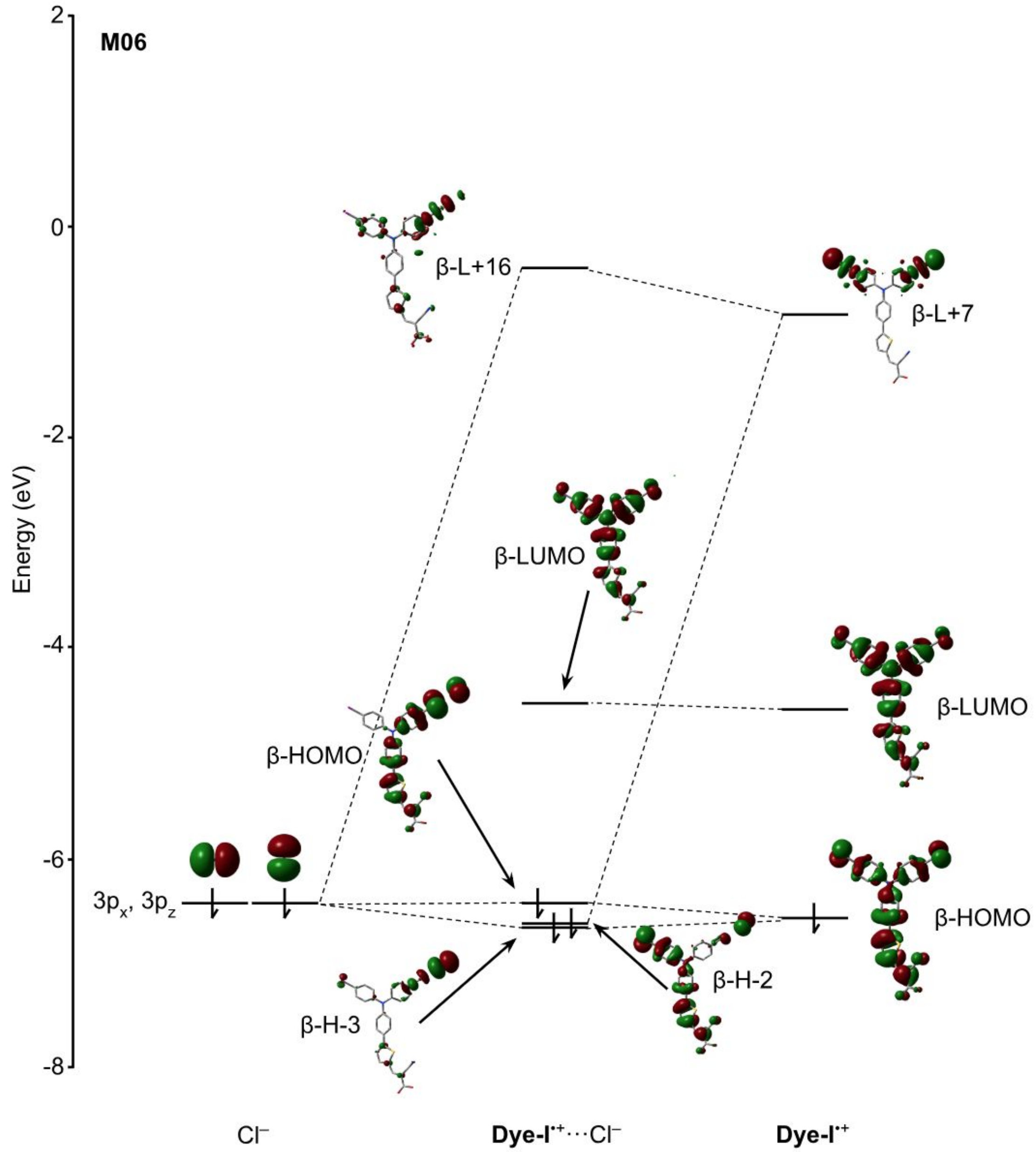

Supplementary Fig. 18 | Dye-I$^{•+}$···Cl^{-} MO mixing diagram with M06. Quantitative MO diagrams were generated of the halogen bonding interaction between **Dye-I$^{•+}$** and Cl^{-}. Only the β-spin single-electron Kohn-Sham orbitals were considered. The eigenvalues for each MO were calculated using M06. These MOs were visualized at an isovalue of 0.002.

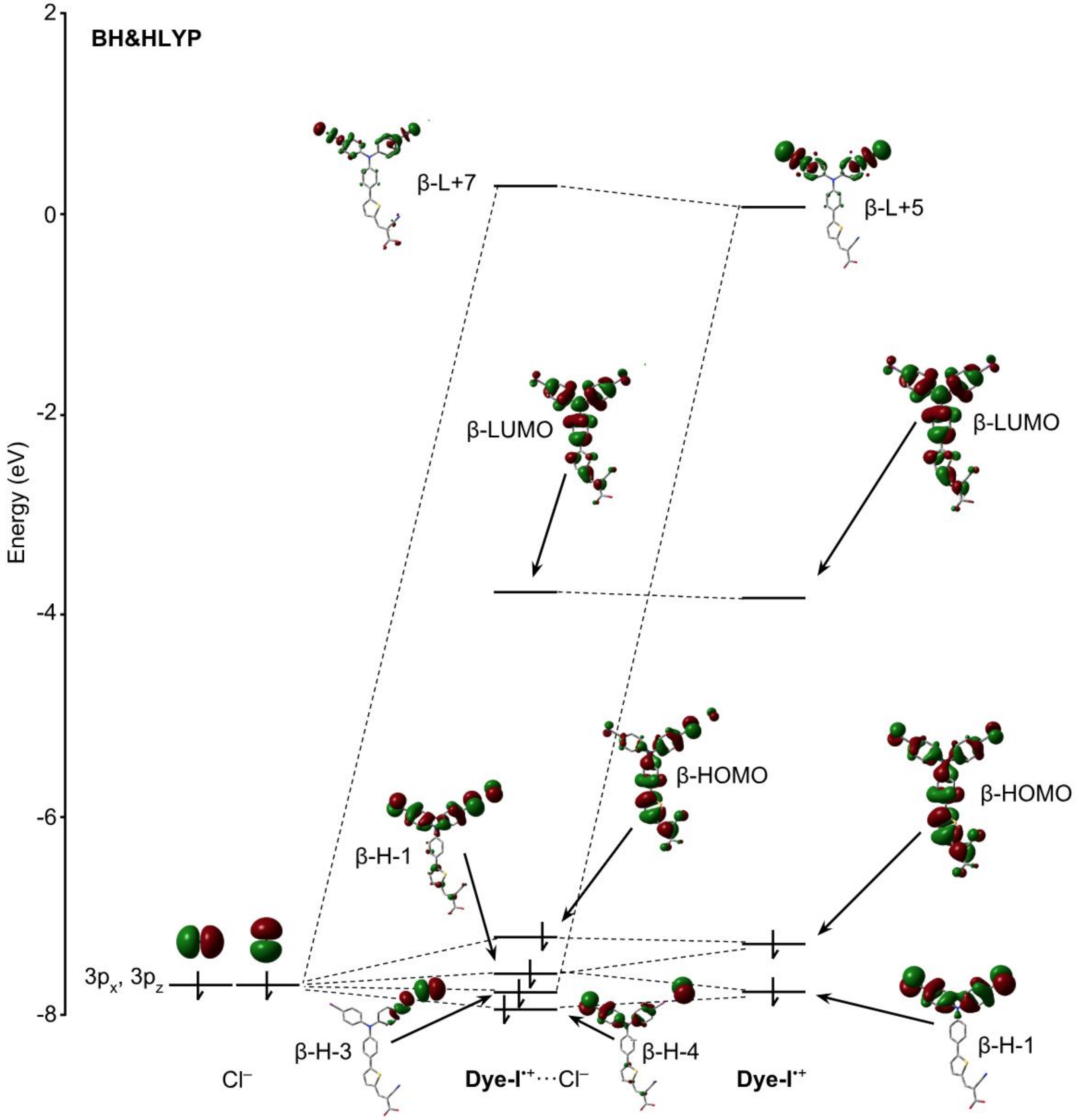

Supplementary Fig. 19 | Dye-I$^{•+}$···Cl^{-} MO mixing diagram with BH&HLYP. Quantitative MO diagrams were generated of the halogen bonding interaction between **Dye-I$^{•+}$** and Cl^{-}. Only the β-spin single-electron Kohn-Sham orbitals were considered. The eigenvalues for each MO were calculated using BH&HLYP. These MOs were visualized at an isovalue of 0.002.

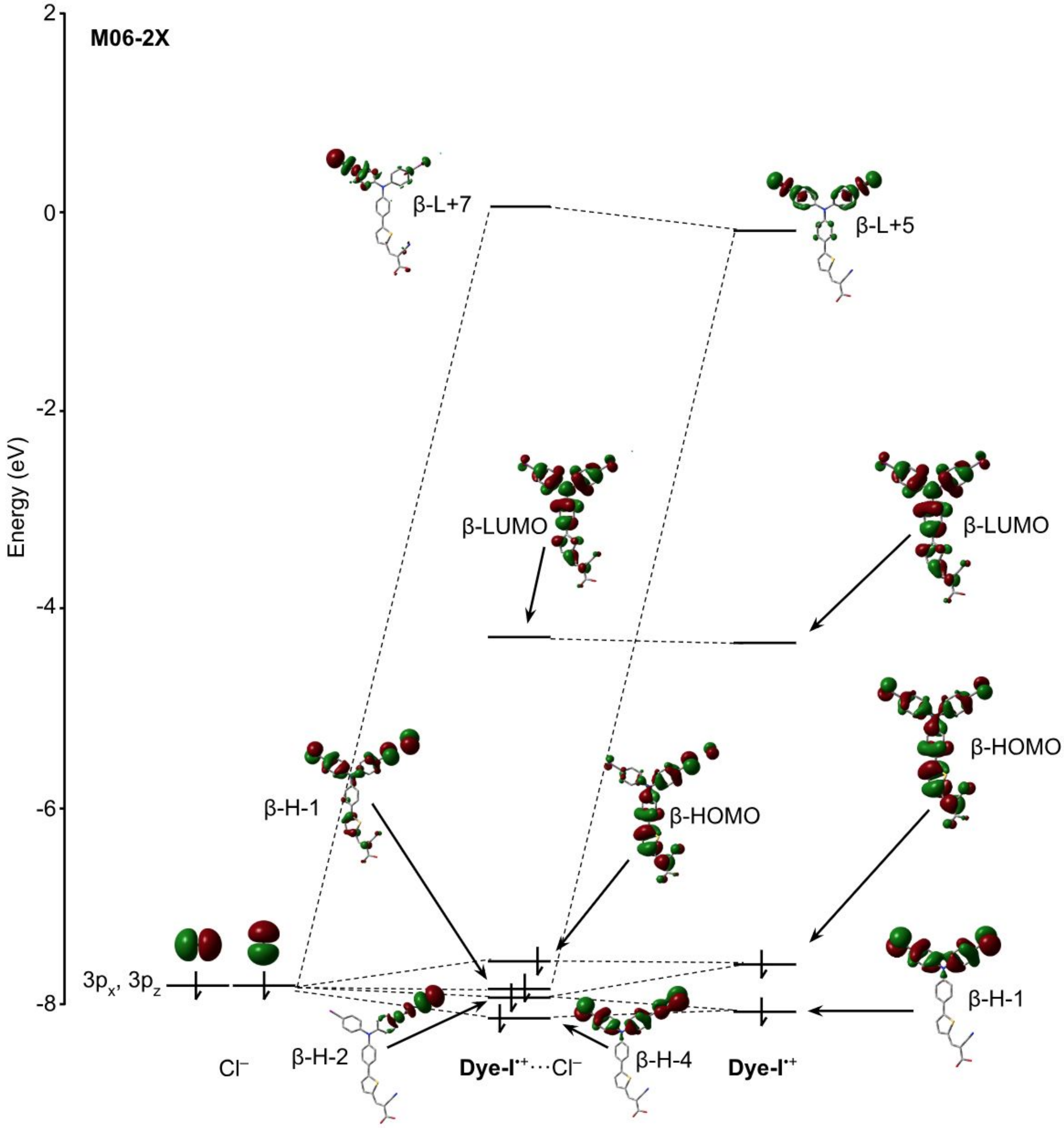

Supplementary Fig. 20 | Dye-I$^{\bullet+}$···Cl$^-$ MO mixing diagram with M06-2X. Quantitative MO diagrams were generated of the halogen bonding interaction between **Dye-I$^{\bullet+}$** and Cl$^-$. Only the β-spin single-electron Kohn-Sham orbitals were considered. The eigenvalues for each MO were calculated using M06-2X. These MOs were visualized at an isovalue of 0.002.

Supplementary Table 1 | Simulated XAS pre-edge parameters for Dye-$I^{\bullet+}\cdots Cl^{-}$.

Method	Hartree-Fock exchange (%)	Pre-edge peak position (eV)[a]	Relative oscillator strength	Cl^{-} 3p orbital mixing coefficient in β-LUMO[b]
Experimental[1]	--	2818.6	--	--
BP86	0	2817.83	1.250	0.01688
M06-L	0	2817.38	0.590	0.00846
O3LYP	11.61	2818.56	0.436	0.00460
B3LYP	20	2818.83	0.226	0.00256
PBE0	25	2818.85	0.182	0.00196
M06	27	2819.60	0.219	0.00240
BH&HLYP	50	2819.86	0.112	0.00073
M06-2X	54	2819.90	0.184	0.00092

[a] Simulated pre-edge positions are corrected from the raw value as described in the Supplementary Methods

[b] Calculated using a Hirshfeld population analysis

Supplementary Table 2 | Simulated XAS pre-edge parameters for Dye-$Br^{\bullet+}\cdots Cl^{-}$.

Method	Hartree-Fock exchange (%)	Pre-edge peak position (eV)[a]	Relative oscillator strength	Cl^{-} 3p orbital mixing coefficient in β-LUMO[b]
Experimental[1]	--	2818.4	--	--
BP86	0	2817.52	1.864	0.02283
M06-L	0	2816.97	0.406	0.00512
O3LYP	11.61	2818.20	0.274	0.00260
B3LYP	20	2818.53	0.106	0.00123
PBE0	25	2818.54	0.081	0.00093
M06	27	2819.27	0.081	0.00090
BH&HLYP	50	2819.61	0.047	0.00038
M06-2X	54	2819.66	0.081	0.00049

[a] Simulated pre-edge positions are corrected from the raw value as described in the Supplementary Methods
[b] Calculated using a Hirshfeld population analysis

Supplementary Table 3 | Simulated XAS pre-edge parameters for Dye-F$^{•+}$···Cl$^{–}$.

Method	Hartree-Fock exchange (%)	Pre-edge peak position (eV)[a]	Relative oscillator strength	Cl$^{–}$ 3p orbital mixing coefficient in β-LUMO[b]
Experimental[1]	--	2818.7	--	--
BP86	0	2817.87	0.703	0.00732
M06-L	0	2817.48	0.157	0.00215
O3LYP	11.61	2818.61	0.208	0.00207
B3LYP	20	2818.89	0.120	0.00147
PBE0	25	2818.88	0.100	0.00123
M06	27	2819.60	0.109	0.00131
BH&HLYP	50	2819.71	0.090	0.00086
M06-2X	54	2819.76	0.141	0.00100

[a] Simulated pre-edge positions are corrected from the raw value as described in the Supplementary Methods
[b] Calculated using a Hirshfeld population analysis

Supplementary Table 4 | Energy correction factors for simulated XAS spectra.

Method	Uncorrected **Dye-Br**$^{•+}$···$Cl^{–}$ edge peak position (eV)	Simulated XAS energy correction (eV)[a]
BP86	2748.78	75.8
M06-L	2760.35	64.3
O3LYP	2757.74	66.9
B3LYP	2769.45	55.2
PBE0	2774.15	50.5
M06	2772.88	51.7
BH&HLYP	2803.06	21.5
M06-2X	2798.56	26.0

[a] Calculated from the difference between the experimental **Dye-Br**$^{•+}$···$Cl^{–}$ edge peak at 2824.6 eV[1] and the uncorrected calculated edge peak.

Supplementary Methods

Quantum-mechanical calculations were carried out using the Gaussian 16 (G16) software package[2] or the ORCA 4.1.1 software package,[3,4] as indicated. All calculations were carried out in SMD modeled acetonitrile.[5] Initial geometry optimizations were carried out in G16 using the unrestricted M06-2X[6,7] functional with an ultrafine integration grid (99 radial shells, 590 angular points) and with aug-cc-pVDZ-PP on iodine, aug-cc-pVDZ on all other halogens, and jun-cc-pVDZ on all other elements.[8–15] All geometries were optimized to a minimum and frequency calculations performed at the same level of theory to verify the absence of imaginary frequencies. The optimized geometric coordinates are reported in Supplemental Data 1. It should be noted that only one conformational isomer was considered in the calculated **Dye-X** compounds. This is not expected to have a significant impact on our conclusions. Single point energy calculations for MO analysis were performed in G16 using the BP86;[16,17] M06-L;[6,7] O3LYP;[18] B3LYP;[19,20] PBE0;[21–23] M06;[6,7] BHandHLYP;[24] or M06-2X[6,7] functionals, as indicated, with an ultrafine integration grid (99 radial shells, 590 angular points) and with aug-cc-pVTZ-PP on iodine, aug-cc-pVTZ on all other halogens, and jun-cc-pVTZ on all other elements.[8–15] Hirshfeld population analysis on the chloride contributions to the frontier MOs was carried out using the Multiwfn 3.4 software package.[25–29] Parameters for the aug-cc-pVDZ-PP and aug-cc-pVTZ-PP basis sets were obtained from the ESML basis set exchange.[30,31]

Except where noted, the XAS spectra were simulated in ORCA using TD-DFT employing a refined integration grid (Grid6: Lebedev = 590, IntAcc = 5.34), the Tamm-Dancoff approximation,[32] the Douglas-Kroll-Hess procedure to correct for scalar relativistic effects,[33–37] and the aug-cc-pVTZ-DK basis set on halogen atoms and jun-cc-pVTZ-DK on all other elements,[8–11,14,38] where the “jun-” diffuse functions are defined using the “calendar” basis set scheme proposed by Truhlar and coworkers.[15] These calculations also made use of the libint2 library for the determination of 2-electron integrals.[39] These calculations were carried out using the same functional series as the single point energy calculations detailed above. The CIS(D) simulation of the [p-iodoaniline]$^{\bullet+}\cdots Cl^{-}$ model system was carried out in ORCA using the orbital optimized MP2 procedure of Neese and Grimme,[40] employing the RI approximation[41,42] supported by automatically generated auxiliary basis sets.[43]

Chlorine K-edge XAS spectral simulations were generated using standard TD-DFT or CIS(D) methodology, except only transitions from the Cl 1s core orbital to virtual orbitals were allowed.[44,45] Both alpha and beta spin transitions were included and quadrupole contributions to the transitions were considered. To ensure that the pre-edge features and the edge-peak were simulated in their entirety, 100 transitions were calculated for OOMP2 and the hybrid functionals and 200 transitions were calculated for the BP86 and M06-L functionals. TD-DFT methods are known to significantly underestimate the energy of XAS transitions as a result of the omission of occupied orbital relaxation in response to the core hole, the omission of relativistic stabilization, and other errors associated with the functional.[45–47] To correct for this underestimation, functional-specific energy shifts were established by adjusting the edge peak calculated for **Dye-Br**$^{\bullet+}\cdots Cl^{-}$ to the position of the experimental edge peak at 2824.6 eV,[1] and these shifts were applied to the calculated **Dye-I**$^{\bullet+}\cdots Cl^{-}$ and **Dye-F**$^{\bullet+}\cdots Cl^{-}$ spectra. The values of these energy shifts are reported in Supplementary Table 4. A similar energy shift was applied the CIS(D)

simulated spectrum of [p-iodoaniline]$^{\bullet+}\cdots Cl^-$. The CIS(D) correction value was determined to be -30.56 eV based on the difference between the CIS(D) calculated $\pi^*\leftarrow Cl_{1s}$ transition of free chloride ion (unrestricted OOMP2 level, 2855.26 eV) and the experimental Cl^- edge peak (2824.7 eV).[1] Spectral simulations were generated using the orca_mapspc utility with an arbitrary 2 eV linewidth applied to all transitions.[3,4] EDDM cube files were generated using the orca_plot utility at a 100 100 100 grid.[3,4]

Supplementary References

1. Parlane, F. G. L. *et al.* Spectroscopic detection of halogen bonding resolves dye regeneration in the dye-sensitized solar cell. *Nat. Commun.* **8**, 1761 (2017).
2. Frisch, M. J. *et al. Gaussian 16, Revision B.01*. (Gaussian Inc., 2016).
3. Neese, F. The ORCA program system. *Wiley Interdiscip. Rev. Comput. Mol. Sci.* **2**, 73–78 (2012).
4. Neese, F. Software update: the ORCA program system, version 4.0. *Wiley Interdiscip. Rev. Comput. Mol. Sci.* **8**, e1327 (2017).
5. Marenich, A. V., Cramer, C. J. & Truhlar, D. G. Universal solvation model based on solute electron density and on a continuum model of the solvent defined by the bulk dielectric constant and atomic surface tensions. *J. Phys. Chem. B* **113**, 6378–6396 (2009).
6. Zhao, Y. & Truhlar, D. G. The M06 suite of density functionals for main group thermochemistry, thermochemical kinetics, noncovalent interactions, excited states, and transition elements: two new functionals and systematic testing of four M06-class functionals and 12 other functionals. *Theor. Chem. Acc.* **120**, 215–241 (2007).
7. Zhao, Y. & Truhlar, D. G. Density functionals with broad applicability in chemistry. *Acc. Chem. Res.* **41**, 157–167 (2008).
8. Dunning, T. H. Gaussian basis sets for use in correlated molecular calculations. I. The atoms boron through neon and hydrogen. *J. Chem. Phys.* **90**, 1007–1018 (1989).
9. Woon, D. E. & Dunning, T. H. Gaussian basis sets for use in correlated molecular calculations. III. The atoms aluminum through argon. *J. Chem. Phys.* **98**, 1358–1315 (1993).
10. Kendall, R. A., Dunning, T. H. & Harrison, R. J. Electron affinities of the first-row atoms

revisited. Systematic basis sets and wave functions. *J. Chem. Phys.* **96**, 6796–6806 (1992).

11. Peterson, K. A., Woon, D. E. & Dunning, T. H. Benchmark calculations with correlated molecular wave functions. IV. The classical barrier height of the $H+H_2 \rightarrow H_2+H$ reaction. *J. Chem. Phys.* **100**, 7410–7415 (1994).
12. Peterson, K. A., Shepler, B. C., Figgen, D. & Stoll, H. On the spectroscopic and thermochemical properties of ClO, BrO, IO, and their anions. *J. Phys. Chem. A* **110**, 13877–13883 (2006).
13. Peterson, K. A., Figgen, D., Goll, E., Stoll, H. & Dolg, M. Systematically convergent basis sets with relativistic pseudopotentials. II. Small-core pseudopotentials and correlation consistent basis sets for the post-d group 16–18 elements. *J. Chem. Phys.* **119**, 11113–11112 (2003).
14. Hashimoto, T., Hirao, K. & Tatewaki, H. Comment on Dunning's correlation-consistent basis sets. *Chem. Phys. Lett.* **243**, 190–192 (1995).
15. Papajak, E., Zheng, J., Xu, X., Leverentz, H. R. & Truhlar, D. G. Perspectives on basis sets beautiful: Seasonal plantings of diffuse basis functions. *J. Chem. Theory Comput.* **7**, 3027–3034 (2011).
16. Perdew, J. P. Density-functional approximation for the correlation energy of the inhomogeneous electron gas. *Phys. Rev. B Condens. Matter* **33**, 8822–8824 (1986).
17. Becke, A. D. Density-functional exchange-energy approximation with correct asymptotic behavior. *Phys. Rev. A Gen. Phys.* **38**, 3098–3100 (1988).
18. Cohen, A. J. & Handy, N. C. Dynamic correlation. *Mol. Phys.* **99**, 607–615 (2001).
19. Lee, C., Yang, W. & Parr, R. G. Development of the Colle-Salvetti correlation-energy formula into a functional of the electron density. *Phys. Rev. B Condens. Matter* **37**, 785–789 (1988).

20. Becke, A. D. Density-functional thermochemistry. III. The role of exact exchange. *J. Chem. Phys.* **98**, 5648–5646 (1993).
21. Perdew, J. P., Burke, K. & Ernzerhof, M. Generalized gradient approximation made simple. *Phys. Rev. Lett.* **77**, 3865–3868 (1996).
22. Perdew, J. P., Burke, K. & Ernzerhof, A. M. Errata: Generalized gradient approximation made simple. *Phys Rev Lett* **78**, 1396 (1996).
23. Adamo, C. & Barone, V. Toward reliable density functional methods without adjustable parameters: The PBE0 model. *J. Chem. Phys.* **110**, 6158–6114 (1999).
24. Becke, A. D. A new mixing of Hartree–Fock and local density-functional theories. *J. Chem. Phys.* **98**, 1372–1377 (1993).
25. Hirshfeld, F. L. Bonded-atom fragments for describing molecular charge densities. *Theor. Chim. Acta* **44**, 129–138 (1977).
26. Ritchie, J. P. Electron density distribution analysis for nitromethane, nitromethide, and nitramide. *J. Am. Chem. Soc.* **107**, 1829–1837 (1985).
27. Ritchie, J. P. & Bachrach, S. M. Some methods and applications of electron density distribution analysis. *J. Comput. Chem.* **8**, 499–509 (1987).
28. Lu, T. & Chen, F. Multiwfn: a multifunctional wavefunction analyzer. *J. Comput. Chem.* **33**, 580–592 (2012).
29. Chen, T. Calculation of molecular orbital composition. *Acta Chimica Sinica* **69**, (2011).
30. Feller, D. The role of databases in support of computational chemistry calculations. *J. Comput. Chem.* **17**, 1571–1586 (1996).
31. Schuchardt, K. L. *et al.* Basis set exchange: a community database for computational sciences. *J. Chem. Inf. Model.* **47**, 1045–1052 (2007).
32. Petrenko, T., Kossmann, S. & Neese, F. Efficient time-dependent density functional theory

approximations for hybrid density functionals: analytical gradients and parallelization. *J. Chem. Phys.* **134**, 054116 (2011).

33. Douglas, M. & Kroll, N. M. Quantum electrodynamical corrections to the fine structure of helium. *Ann. Phys.* **82**, 89–155 (1974).
34. Hess, B. A. Applicability of the no-pair equation with free-particle projection operators to atomic and molecular structure calculations. *Phys. Rev. A Gen. Phys.* **32**, 756–763 (1985).
35. Hess, B. A. Relativistic electronic-structure calculations employing a two-component no-pair formalism with external-field projection operators. *Phys. Rev. A Gen. Phys.* **33**, 3742–3748 (1986).
36. Reiher, M. & Wolf, A. Exact decoupling of the Dirac Hamiltonian. II. The generalized Douglas-Kroll-Hess transformation up to arbitrary order. *J. Chem. Phys.* **121**, 10945–10956 (2004).
37. Jansen, G. & Hess, B. A. Revision of the Douglas-Kroll transformation. *Phys. Rev. A Gen. Phys.* **39**, 6016–6017 (1989).
38. de Jong, W. A., Harrison, R. J. & Dixon, D. A. Parallel Douglas–Kroll energy and gradients in NWChem: Estimating scalar relativistic effects using Douglas–Kroll contracted basis sets. *J. Chem. Phys.* **114**, 48–53 (2001).
39. Valeev, E. F. *LIBINT: a library for the evaluation of molecular integrals of many-body operators over Gaussian functions*.
40. Neese, F., Schwabe, T., Kossmann, S., Schirmer, B. & Grimme, S. Assessment of orbital-optimized, spin-component scaled second-order many-body perturbation theory for thermochemistry and kinetics. *J. Chem. Theory Comput.* **5**, 3060–3073 (2009).
41. Feyereisen, M., Fitzgerald, G. & Komornicki, A. Use of approximate integrals in ab initio theory. An application in MP2 energy calculations. *Chem. Phys. Lett.* **208**, 359–363 (1993).

42. Kendall, R. A. & Früchtl, H. A. The impact of the resolution of the identity approximate integral method on modern ab initio algorithm development. *Theor. Chem. Acc.* **97**, 158–163 (1997).

43. Stoychev, G. L., Auer, A. A. & Neese, F. Automatic Generation of Auxiliary Basis Sets. *J. Chem. Theory Comput.* **13**, 554–562 (2017).

44. DeBeer George, S., Petrenko, T. & Neese, F. Time-dependent density functional calculations of ligand K-edge X-ray absorption spectra. *Inorg. Chim. Acta* **361**, 965–972 (2008).

45. Ray, K., Debeer George, S., Solomon, E. I., Wieghardt, K. & Neese, F. Description of the ground-state covalencies of the bis(dithiolato) transition-metal complexes from X-ray absorption spectroscopy and time-dependent density-functional calculations. *Chemistry* **13**, 2783–2797 (2007).

46. Kozimor, S. A. *et al.* Trends in covalency for d- and f-element metallocene dichlorides identified using chlorine K-edge X-ray absorption spectroscopy and time-dependent density functional theory. *J. Am. Chem. Soc.* **131**, 12125–12136 (2009).

47. Minasian, S. G. *et al.* Determining relative f and d orbital contributions to M-Cl covalency in $MCl_6^{(2-)}$ (M = Ti, Zr, Hf, U) and $UOCl_5^{(-)}$ using Cl K-edge X-ray absorption spectroscopy and time-dependent density functional theory. *J. Am. Chem. Soc.* **134**, 5586–5597 (2012).
