# Supplementary material for: π covalency in the halogen bond
Source: Nat Commun. 2020 Jul 3;11:3310. doi: 10.1038/s41467-020-17122-7 (PMC7335087; doi:10.1038/s41467-020-17122-7)
Supplement: Supplementary file 2 — Description of Additional Supplementary Files [file 41467_2020_17122_MOESM2_ESM.pdf]

Description of Additional Supplementary Files

File name: Supplementary Data 1
Description: Supplementary Data 1 contains geometry optimized molecular coordinates and single-point energies for all DFT models used in this study. All molecular geometries were optimized using the M06-2X functional as described in the Supplemental Methods and the single-point energies reported here were calculated at the same level of theory.
